# A Comprehensive Review of Data-Driven Techniques for Air Pollution Concentration Forecasting

**DOI:** 10.3390/s25196044

**Published:** 2025-10-01

**Authors:** Jaroslaw Bernacki, Rafał Scherer

**Affiliations:** 1 Department of Computer Science and Engineering Systems, Wrocław University of Science and Technology, Wyb. Wyspiańskiego 27, 50-370 Wrocław, Poland; 2 Department of Artificial Intelligence, Czȩstochowa University of Technology, al. Armii Krajowej 36, 42-200 Czȩstochowa, Poland; rafal.scherer@pcz.pl; 3 Faculty of Computer Science, AGH University of Kraków, 30-059 Kraków, Poland

**Keywords:** air pollution, forecasting, machine learning, deep models, particulate matter

## Abstract

Air quality is crucial for public health and the environment, which makes it important to both monitor and forecast the level of pollution. Polluted air, containing harmful substances such as particulate matter, nitrogen oxides, or ozone, can lead to serious respiratory and circulatory diseases, especially in people at risk. Air quality forecasting allows for early warning of smog episodes and taking actions to reduce pollutant emissions. In this article, we review air pollutant concentration forecasting methods, analyzing both classical statistical approaches and modern techniques based on artificial intelligence, including deep models, neural networks, and machine learning, as well as advanced sensing technologies. This work aims to present the current state of research and identify the most promising directions of development in air quality modeling, which can contribute to more effective health and environmental protection. According to the reviewed literature, deep learning–based models, particularly hybrid and attention-driven architectures, emerge as the most promising approaches, while persistent challenges such as data quality, interpretability, and integration of heterogeneous sensing systems define the open issues for future research.

## 1. Introduction

Air quality is crucial for human health and the functioning of ecosystems. Polluted air contains harmful substances such as particulate matter (PM), nitrogen oxides, or benzo(a)pyrene, which can cause serious respiratory and circulatory system diseases [1]. Long-term exposure to pollution increases the risk of asthma, chronic obstructive pulmonary disease (COPD), and cancer [2,3]. Children, the elderly, and people suffering from chronic diseases are particularly at risk [4]. Monitoring air quality allows for early warning of smog and taking action to reduce the emission of harmful substances. Thanks to appropriate regulations and initiatives, such as low-emission zones or the development of electric transport, air quality can be effectively improved. Taking care of clean air is an investment in the health of society and environmental protection [5].

The monitoring of air quality includes predicting the concentrations of various pollutants that have a significant impact on human health and the environment. The most frequently forecasted are particulate matter PM_10_ and PM_2.5_, which, due to their small diameter, can penetrate the respiratory and circulatory systems, causing serious health problems [6]. Gases such as sulfur dioxide (${\mathrm{SO}}_{2}$), carbon monoxide (CO), and nitrogen oxides (NO and ${\mathrm{NO}}_{2}$), which mainly come from the combustion of fossil fuels and road traffic, are also an important element of forecasts [7]. Particular attention is also paid to ozone ($O_{3}$), which is formed as a result of chemical reactions in the atmosphere and can be harmful at high concentrations. Depending on local meteorological conditions and emission sources, forecasts may also include other substances, such as benzo(a)pyrene or volatile organic compounds (VOCs). Indicators such as the Air Quality Index (AQI) [8] and the Air Pollution Index (API) [9] are used to classify air quality and inform the public about the level of risk. AQI used worldwide takes into account various pollutants and presents the results on an easy-to-read numerical and color scale, while API is an older indicator, used mainly in some Asian countries. There are also other metrics, such as AQHI [10], CAQI [11], DAQI [12], CAI [13], PSI [8], IAQI [14], which are used in different countries. A summary of the air quality indicators is presented in Table 1.

**Figure 1:**
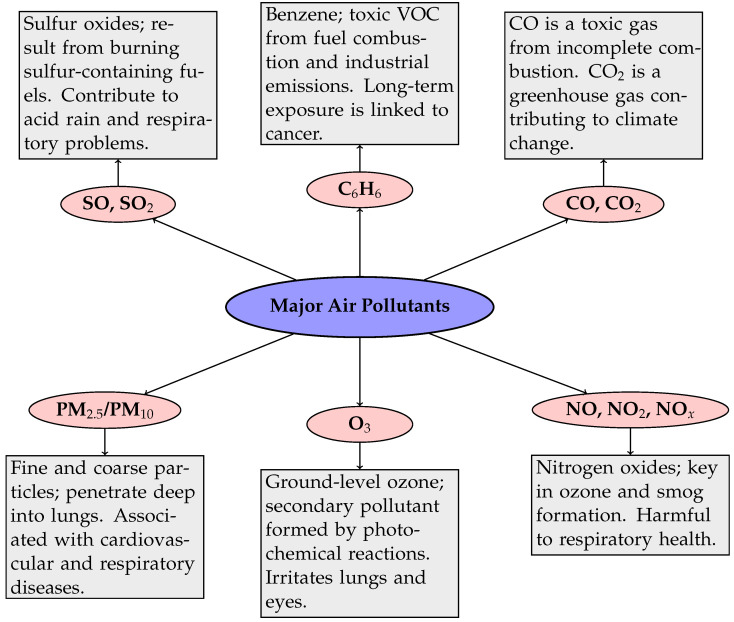
Major air pollutants and their short descriptions [16].

Information on predicted air quality also helps in planning environmental policy, e.g., limiting car traffic or introducing smog alerts. Modern methods based on artificial intelligence and numerical models allow for increasingly accurate analysis and forecasting of changes in air quality. The better the forecasts, the more effective preventive measures, which translates into improved public health and environmental protection. Therefore, the development and improvement of air quality forecasting methods should be one of the priorities in environmental policy [15]. The major air pollutants and their short descriptions are described in Figure 1.

**Figure 2:**
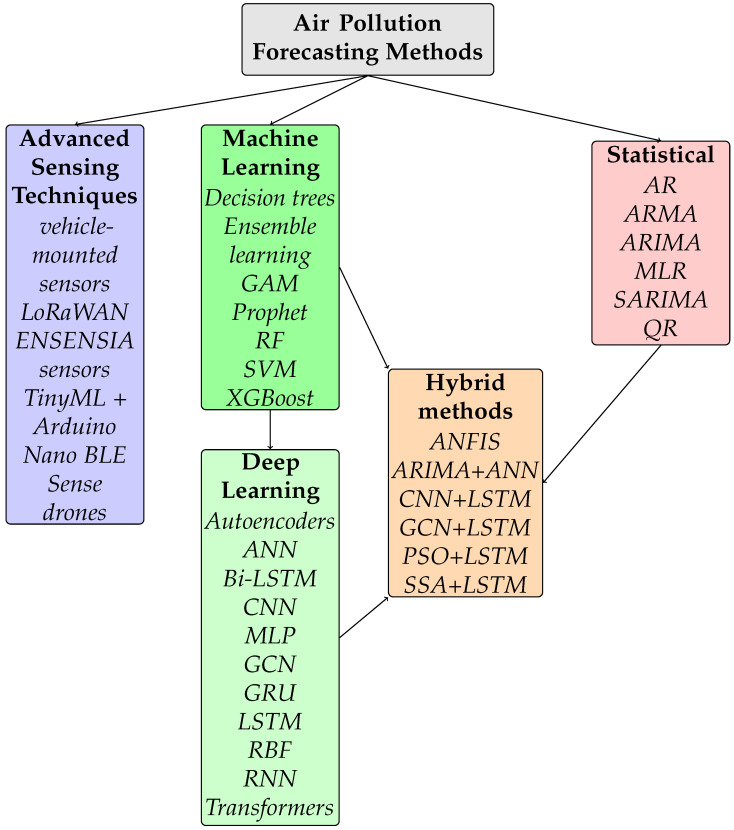
Categorization of forecasting methods with selected algorithms/methods. Deep learning is a subcategory of machine learning [16].

In this paper, we review air quality forecasting methods, including modern techniques based on artificial intelligence, as well as classical approaches based on statistical foundations. In particular, we analyze deep models, such as neural networks, which can effectively predict pollution levels. We also present machine learning methods that use historical data and environmental factors to forecast air quality in various conditions. Additionally, we discuss statistical approaches that are still used in trend analysis and short-term forecasting. Moreover, we analyze advanced sensing technologies, including low-cost, mobile, and IoT-based air quality monitoring systems, which enable flexible and scalable data acquisition. These sensor-based solutions, when properly calibrated and enhanced with AI, can complement traditional monitoring networks and significantly improve data granularity. We analyze the efficacy of these methods, indicating their advantages and limitations in the context of different forecasting scenarios. Therefore, the goal of the paper is to present the current state of research and indicate the most promising directions of development in the field of air quality modeling and monitoring. The relations of forecasting methods discussed in the paper are presented in Figure 2.

Organization of the paper and conventions

The paper adopts the structure of a traditional narrative review and is organized as follows. In Section 2, we describe the advanced sensing techniques for air pollution monitoring and detection. Section 3 describes neural networks and deep learning models. In Section 4, the description of classical machine learning methods is included. In the Section 5, the statistical methods for forecasting are described. Section 6 presents the hybrid methods. Each section is summarized in a table and a discussion subsection, which contains all the methods discussed. In Section 7, other methods for forecasting are considered. Section 8 summarizes the paper, with the directions of open issues and future work.

At the end of the paper, there is an appendix that presents full explanations of the abbreviations of the forecasting methods reviewed in this survey. Additionally, it includes formulas for all quality measures used in the analysis, which facilitates their interpretation and a comparison of results.

## 2. Advanced Sensing Techniques

This section presents an overview of air pollution detection methods using various types of sensors, both stationary and mobile. Technologies based on low-cost sensors, drones, and wireless sensor networks are discussed. The advantages and limitations of each solution are also highlighted in terms of measurement accuracy, weather resistance, and integration with modern data processing technologies.

In [17], a low-cost LoRaWAN wireless network for particulate matter (PM) measurements, tested in an industrial setting (Riotinto mine), is developed. The devices are based on SDS011 sensors and Arduino and Raspberry Pi components. Local calibration was performed, achieving an MAE of less than 0.3 μg/m^3^ and a coefficient of determination of $R^{2}=0.96$. The network enables real-time data collection and transmission to a cloud platform. The authors point to the solution’s high cost-effectiveness for environmental monitoring.

The aim of the study [18] was to calibrate a low-cost ${\mathrm{NO}}_{2}$, NO, $O_{3}$, CO, and PM_2.5_ sensors called ENSENSIA in an urban environment using machine- and deep learning models. Data collected in a German city were compared with official reference stations. After calibration, high agreement was achieved ($R^{2}$ > 0.86) and measurement error was reduced. The authors demonstrate that onsite calibration significantly improves the reliability of measurements. The study emphasizes the need for dynamic correction of low-end sensors.

The paper [19] describes the design of a low-cost drone-mounted air quality measurement system for the city of Opole, Poland. The system utilizes a PMS7003 sensor for PM_2.5_ and PM_10_ detection, MQ sensors for gas detection (${\mathrm{NO}}_{2}$, CO), and a GPS module. The total cost of the device was under EUR 70, making it extremely competitive. The results showed that the UAV-derived data correlated with ground-based data. The paper demonstrates the potential of drones for the rapid and cost-effective identification of smog-prone areas.

In [20], the drones equipped with laser sensors for detecting gaseous pollutants ($C_{6}H_{6}$, HCHO, ${\mathrm{SO}}_{2}$); and particulate matter (PM_1_, PM_2.5_, and PM_10_) and GPS sensors to monitor linear emissions (e.g., along roads and railways) were used. The devices analyzed pollutants in the vertical profile of the atmosphere and along transportation routes. The drones operated autonomously and collected data under variable atmospheric conditions. The results were compared with data from reference stations. This work suggests that UAVs are the future of microregional emission source detection.

In [21], the Enviro-IoT system, which is a low-cost, real-time air quality monitoring solution based on miniature sensors and IoT technology, is presented. The authors describe the four-year design and implementation process of the system, then present the results of a nine-month study in urban environments. The system was compared with industrial-grade testing equipment and achieved high measurement accuracy for PM_2.5_, PM_10_, and ${\mathrm{NO}}_{2}$ (98%, 97%, and 97%, respectively). The analysis included over 57,000 samples, confirming the solution’s effectiveness. The paper demonstrates that low-cost sensors combined with IoT can be an effective alternative to traditional measurement systems.

The research [22] involves developing a low-cost, self-built air quality assessment system that can be used both indoors and outdoors. The system is based on ESP32 microcontrollers using the ESP-NOW wireless communication protocol and Alphasense sensors to measure the concentration of aerosols and gases such as ${\mathrm{NO}}_{2}$, ${\mathrm{SO}}_{2}$, $O_{3}$, CO, ${\mathrm{CO}}_{2}$, and $H_{2}S$. The device was used to assess air contaminated with tobacco smoke after passing through a DBD plasma purification reactor. A significant reduction in aerosol and ${\mathrm{SO}}_{2}$ particles was observed, while an undesirable increase in ${\mathrm{NO}}_{2}$ was observed. The measurement results were consistent with trends observed with the professional GRIMM device, confirming the potential of the developed system as a cost-effective and effective alternative to air quality monitoring.

The work [23] focuses on developing a universal method for calibrating low-cost air quality sensors, enabling their mass deployment in urban monitoring networks. Standard approaches require individual calibration of each sensor, which generates high costs and limits scalability. The authors propose a global calibration method without transfer samples, based on data from a limited number of sensors and machine learning algorithms, that can be applied to all devices of a given type. Tests conducted at different times of the year have shown that the effectiveness of this method is comparable to approaches requiring individual calibration parameters. Implementing such calibration could significantly reduce costs and enable the widespread use of accurate, low-cost IoT devices for air quality monitoring.

The study [24], conducted in Delhi, examined three popular PM sensor models (SPS30, PMS7003, HPMA115C0-004) in high humidity and dust conditions for PM_2.5_ detection. The work highlights the need for dynamic calibration and the implementation of real-time humidity compensation. Long-term sensor drift analysis is also conducted.

In [25], the application of TinyML platforms to real-time ozone prediction using low-cost devices such as the Arduino Nano BLE Sense is demonstrated. The aim of the system is to detect the CO concentrations, using temperature and air pressure. The solution is low-cost and operates in real time, and can be mounted on drones.

In [26], the calibration of low-cost particulate matter sensors, which are gaining popularity due to their low price and portability, is addressed. Standard calibration methods require large amounts of data from simultaneous reference measurements, which is time-consuming and expensive. The authors propose the use of transfer learning methods, which allow for efficient sensor calibration with minimal interaction time with the reference device. In particular, they present a meta-learning approach that utilizes data from other sensors and a limited amount of data from the target sensor. Experiments on PM_2.5_ concentrations show that this method outperforms other compared approaches in terms of calibration efficiency.

The research presented in [27] proposes the creation of high-resolution PM_2.5_ maps by combining data from 320 mobile vehicle-mounted sensors and 52 stationary stations. Machine learning algorithms were used for data correction and spatial interpolation. The maps achieved a resolution of 500 m and a 5-min refresh rate, with a global error of +4.3%. The work demonstrates the enormous potential of hybrid (mobile-stationary) systems for managing urban air quality.

The paper [28] presents a low-cost, multi-parameter air quality monitoring system that utilizes various machine learning algorithms for calibration. The system simultaneously measures the concentration of particulate matter (PM_2.5_, PM_10_) and gases (${\mathrm{SO}}_{2}$, ${\mathrm{NO}}_{2}$, CO, $O_{3}$), taking into account temperature and humidity. A prediction model was developed and compared with data from reference devices, evaluating the performance of different ML algorithms. The results demonstrate high prediction accuracy ($R^{2}$ up to 0.99) with low RMSE and MAE errors. It was demonstrated that the system supported by ML algorithms can effectively predict the concentration of particulate matter and gases in the air.

The paper [29] presents a low-cost air quality monitoring platform based on an unmanned aerial vehicle (UAV) equipped with specialized sensors for measuring CO, $O_{3}$, and ${\mathrm{NO}}_{2}$. The solution aims to increase the mobility and availability of measurements in hard-to-reach locations, addressing the limitations of traditional measurement networks, such as high costs and a lack of mobility. The system enables real-time data collection and UAV tracking using LoRa communication and the PTECA web application. The platform provides data visualization and live monitoring during sample collection. Initial tests in controlled airspace using geofencing confirmed the effectiveness and practicality of the proposed solution.

The advanced sensing techniques are summarized in Table 2.

### Discussion

Recent advances in low-cost air quality monitoring systems demonstrate remarkable diversity in sensing platforms, sensor types, and data processing techniques. Many studies emphasize mobile and flexible deployment, such as drone-based platforms and vehicle-mounted sensors, which offer high spatial resolution in dynamic environments. Fixed IoT-based networks like Enviro-IoT or LoRaWAN provide real-time coverage at low cost. A common theme is the need for calibration, either via on-site methods or via scalable machine learning solutions. Overall, modern systems combine compact sensors with machine learning methods, wireless communication (e.g., ESP-NOW, LoRa), and sometimes TinyML, enabling efficient and accurate air pollution detection across various environments.

In addition to low-cost and mobile sensing platforms, a wide range of sensor types is employed in air quality monitoring, each offering distinct advantages and limitations. IoT-enabled low-cost devices provide high-density local coverage, while reference-grade ground stations remain the standard for accuracy and regulatory compliance. Satellite-based imaging systems contribute large-scale spatial coverage and long-term historical records, although with coarser temporal resolution. Aircraft- and drone-mounted sensors fill the gap by delivering flexible, high-resolution observations in areas that are otherwise inaccessible. Together, these complementary approaches highlight the importance of sensor diversity in environmental monitoring, as no single technology alone can provide the accuracy, coverage, and scalability required for comprehensive air quality forecasting.

However, despite these advances, low-cost sensors remain prone to drift, humidity sensitivity, and degradation over time, which can compromise data reliability; in this context, machine learning models are particularly valuable because they can dynamically learn correction patterns, compensate for non-linear sensor behavior, and extend the practical usability of such systems. Low-cost solutions provide significant flexibility, mobility, and scalability, making them attractive for dense urban deployments; however, their effectiveness is often constrained by challenges such as long-term stability, susceptibility to atmospheric conditions (e.g., humidity and temperature fluctuations), signal drift, and the limited lifetime of inexpensive components. Although local calibration procedures and ML algorithms can decrease some of these shortcomings, persistent issues related to standardization, interoperability, power quality, and data transmission limit their widespread adoption in industrial or institutional contexts.

By contrast, satellite-based and conventional ground monitoring stations offer higher measurement accuracy and broader spatial coverage but are associated with high costs, limited accessibility, and lower temporal resolution. These differences highlight the importance of data quality and diversity, as relying on a single type of monitoring source can lead to biased or incomplete forecasts. Future research directions should therefore focus on designing robust fusion strategies that integrate heterogeneous datasets from multiple sensor types, exploiting their complementary strengths to achieve improved forecasting accuracy, resilience to missing or noisy data, and broader applicability across different regions and time scales. In particular, hybrid frameworks that combine high-resolution local sensor data with the wide coverage of satellite observations may provide a more holistic representation of pollution dynamics and support more effective environmental decision-making. Moreover, integrating heterogeneous data sources, such as IoT-based networks, mobile monitoring platforms, and satellite observations, could further enhance model generalizability and robustness, ensuring that forecasting systems remain accurate across diverse environments and evolving pollution scenarios.

## 3. Neural Networks and Deep Learning Models

In this section, we present methods for forecasting air pollution concentrations based on deep learning. Modern neural networks, thanks to their ability to model complex nonlinear relationships, show high efficiency in predicting changes in air quality. We will discuss various network architectures, i.e., artificial neural networks (ANN), multilayer perceptron (MLP), convolutional neural networks (CNN), long short-term memory, recurrent neural networks (RNN), which are used in the analysis of time series of pollution.

### 3.1. Artificial Neural Networks

The paper [30] uses the artificial neural network method and the group data handling method to predict CO_2_ emissions based on energy sources. The experiments used data from the following five countries: Iran, Kuwait, Qatar, Saudi Arabia, and the UAE. The evaluation by using the $R^{2}$ confirmed its efficacy in predicting ${\mathrm{CO}}_{2}$ emissions.

In [31], an artificial neural network-based integrated model to predict the indoor environmental parameters, including predicted mean vote and concentrations of ${\mathrm{CO}}_{2}$, PM_10_, and PM_2.5_, in a school building was proposed. The model was optimized using a multi-objective genetic algorithm and prevents spurious regression by excluding irrelevant input variables. The prediction accuracy was evaluated with root mean square error, which demonstrated high accuracy and applicability for building environment control and energy optimization.

The research [32] evaluates the ability of an artificial neural network to predict hourly air pollutant concentrations and air quality indices for Ahvaz (Iran), one of the most polluted cities due to dust storms. The proposed model, using meteorological data, pollutant concentrations, and time-related factors, showed a higher correlation coefficient (R) and a lower root mean square error (RMSE) for six criteria pollutants, compared with other models.

In the paper [33], neural networks combined with meteorological data were used to predict hourly PM_2.5_ concentrations. The experiments were conducted using data from the Beijing Aotizhongxin monitoring station. The proposed method demonstrated high prediction accuracy, achieving better $R^{2}$ values compared to other approaches.

The study in [34] applied an artificial neural network to forecast PM_2.5_ concentrations in Liaocheng. The model, consisting of 11 input parameters, a hidden layer with 6 neurons, and a single output, was trained on 80% of the dataset and validated on 10%. The analysis revealed a gradual decline in PM_2.5_ levels, with the Bayesian Regularization algorithm providing the most accurate predictions and yielding high correlation coefficients. These results demonstrate the ability of ANN to capture nonlinear relationships and to effectively predict monthly PM_2.5_ concentrations. In addition, [35] reported the use of ANN for forecasting hourly PM_2.5_ and PM_10_ levels in Chongqing.

The study in [36] explored the application of artificial neural networks (ANN) and wavelet transform neural networks (WANN) for forecasting daily PM_2.5_ concentrations in Shanghai. Between 2014 and 2020, PM_2.5_ levels declined by 39.3%. The models utilized meteorological variables and pollutant concentrations from previous days, with minimum and maximum temperature and maximum atmospheric pressure identified as the most influential factors. Among the tested models, WANN with the tansig–purelin activation function consistently outperformed ANN at all stages of evaluation, demonstrating high predictive accuracy and confirming its suitability for PM_2.5_ forecasting in support of air quality management strategies.

In [37], an artificial neural network model using meteorological and PM data to predict daily concentrations of PM_2.5_ and PM_10_ is considered. Among the four tested models, the wavelet-based multilayer perceptron neural network outperformed the others, especially when utilizing lagged wind speed as a predictor. The proposed model achieved high accuracy, with $R^{2}$ values up to 0.91 for PM_2.5_ and 0.98 for PM_10_, and low RMSE values. These findings demonstrate the model’s potential for reliable air quality forecasting and issuing timely health alerts.

In [38], an artificial neural network model to forecast short-term ${\mathrm{CO}}_{2}$ emissions in the cereal sector of Apulia (Southern Italy) is proposed. The model aims to evaluate the environmental impact of adopting sustainable farming methods on pollution reduction. The approach offers a flexible alternative to classical methods, which are often too rigid for modeling complex environmental phenomena. The goal is to explore how more eco-friendly agricultural practices can help reduce ${\mathrm{CO}}_{2}$ emissions in the region.

The research in [39] explores machine learning-based prediction of nitrogen dioxide (${\mathrm{NO}}_{2}$) concentrations, utilizing historical pollutant levels and meteorological data. Focusing on Erfurt (Germany), the study uses temporal dependencies through embedded variables, enabling the model to account for local factors such as traffic and special events. Additionally, the approach utilizes pollutant seasonality and seven meteorological parameters to predict ${\mathrm{NO}}_{2}$ levels in the upcoming hours. Experimental results demonstrate higher forecasting accuracy compared to other models, particularly during disruptions in typical seasonal patterns, such as holidays. These predictions can support emission management strategies, including traffic regulation.

### 3.2. Convolutional Neural Networks

In [40], convolutional neural network models were employed to predict real-time PM_10_ and PM_2.5_ concentrations in Poland. The evaluation, based on $R^{2}$ and RMSE measures, confirmed the models’ high accuracy in forecasting the analyzed concentrations.

The paper [41] presents a deep learning model for forecasting PM_2.5_ air quality, addressing the challenges posed by the nonlinear and dynamic nature of multivariate air quality time series data. The proposed model integrates one-dimensional convolutional neural networks (1D-CNNs) to capture local trends and spatial correlations, along with bi-directional long short-term memory networks (Bi-LSTM) to learn spatial–temporal dependencies. This architecture is designed to extract shared features from multivariate time series data related to air quality. Extensive experiments with real-world datasets demonstrate that the model achieves high accuracy in PM_2.5_ forecasting, making it a promising approach for air pollution prediction and management.

In [42], the prediction of particulate matter concentrations in Beijing (China), using deep learning methods, is considered. The paper utilizes a convolutional neural network and a long short-term memory network model to predict hourly PM concentrations, utilizing meteorological data, historical pollutant data, and concentrations from nearby stations as spatial–temporal features. The performance of various deep learning algorithms, including LSTM, Bi-LSTM, GRU, Bi-GRU, and CNN, was compared, with the CNN-LSTM model outperforming all others in predictive accuracy. These results demonstrate the effectiveness of combining convolutional neural networks and long short-term memory networks for air quality prediction.

The work [43] developed a model combining convolutional neural networks and long short-term memory networks for air pollution prediction using sensor data. It considered both univariate and multivariate models, with the latter using data from multiple pollutants and meteorological factors. The model was tested using data from Barcelona, Kocaeli, and Istanbul, showing significant improvements in prediction accuracy compared to traditional LSTM models. Transfer learning was also applied, with better results when transferring the model from Kocaeli to Istanbul, highlighting the model’s adaptability to different cities.

The study [44] proposed a spatiotemporal convolutional neural network and long short-term memory model to predict daily average PM_2.5_ concentrations in Beijing City (China). The model used both linear and nonlinear correlations between target and observation parameters, using historical air quality and meteorological data from 384 monitoring stations across China. By efficiently extracting features through CNN and reflecting long-term data trends with LSTM, the model achieved accurate and fast predictions. Compared to multilayer perceptron and LSTM models, the CNN-LSTM method demonstrated better stability and performance in air quality prediction.

The study presented in [45] introduces a deep learning model that combines a convolutional neural network with a long short-term memory network to predict hourly total suspended particle (TSP) concentrations. The CNN serves as a data processor, utilizing feature extractors based on statistically significant lagged variables. The LSTM network utilizes a new feature mapping scheme to predict the next hourly TSP concentration. Extensive testing of the proposed model demonstrates its better performance compared to an ensemble of five other machine learning models.

In [46], a CNN-LSTM model is developed to forecast the PM_2.5_ concentration for the next 24 h in Beijing. This model combines the strengths of both convolutional neural networks and long short-term memory networks: CNN efficiently extracts air quality-related features, while LSTM captures the long-term temporal context of the data. The model takes air quality data from the past 7 days as input and predicts the next day’s PM_2.5_ concentration. The study compares four models: univariate LSTM, multivariate LSTM, univariate CNN-LSTM, and multivariate CNN-LSTM. The results indicate that the proposed multivariate CNN-LSTM model outperforms the others, achieving the best performance with low error and short training time.

### 3.3. Attention-Based Methods

In the work [47], the authors propose a model combining a wind-sensitive attention mechanism, an LSTM network, and XGBoost to predict PM_2.5_ concentrations, taking into account the influence of wind direction and speed on pollutant transport. The input data included PM_2.5_ measurements from neighboring areas and meteorological forecasts. Experiments were conducted using real PM_2.5_ data and weather forecasts.

The research [48] proposes a new air quality forecasting model based on the IMDA-VAE architecture, which combines a variational autoencoder and an attention mechanism. The method is experimentally validated using pollution data from four US states. Evaluation based on six statistical metrics demonstrates that the model effectively predicts concentrations of various pollutants at multiple locations. The results show that IMDA-VAE outperforms existing models, including LSTM, GRU, and their attention-based versions.

The authors of [49] developed a new air quality forecasting model based on the Bi-LSTM convolutional autoencoder with attention. The study was conducted in Busan (South Korea), using data on particulate matter concentration, meteorological conditions, and road traffic volume obtained using the YOLO algorithm. The model effectively captured spatial–temporal relationships and significantly outperformed previous approaches in both short- and long-term forecasts. Furthermore, it was successfully deployed in the cloud, enabling real-time air quality forecasting.

In [50], an advanced Seq2Seq model, called the attention-based air quality forecasting model (ABAFM), which replaces the RNN encoder with a pure attention mechanism with positional embedding is proposed. This modification reduces training time and enhances the robustness of the model. Experiments conducted at Olympic Center and Dongsi monitoring stations in Beijing show that ABAFM outperforms existing methods, particularly in predicting sudden changes in PM_2.5_ levels.

In the study [51], an image-based deep learning model combining a 3D-CNN, GRU, and attention mechanism to forecast air quality is proposed. The 3D-CNN extracts spatial features from images, the GRU captures temporal dependencies, and the attention mechanism adjusts feature influence to reduce fluctuations. Tested on the Shanghai scenery dataset with air quality data, the model outperformed other state-of-the-art methods. It provides accurate multi-horizon predictions and reliable early warnings for air pollutants.

### 3.4. Multilayer Perceptron

The paper [52] examines the forecasting performance of multiple models for predicting PM_10_ concentrations on the East Coast of Peninsular Malaysia, where PM_10_ is a significant air quality concern. The research compares a linear model (multiple linear regression) with two non-linear models (multilayer perceptron and radial basis function) using meteorological and pollutant historical data at urban, suburban, and rural sites. The non-linear radial basis function model outperforms the linear model, reducing forecasting errors by 78.9% in urban areas, 32.1% in suburban areas, and 39.8% in rural areas. The results highlight the complexity of the relationship between PM_10_ and its contributing factors, with the non-linear models, particularly artificial neural networks, offering more accurate forecasts. This model provides reliable next-day PM_10_ concentration predictions, useful for early warning purposes.

In the work [53], forecasting maximum $O_{3}$ concentrations in urban microlocations is addressed. It proposes an integrated modeling approach using multilayer perceptron neural networks, which combine data from the QualeAria air quality model, WRF weather prediction model, and local air pollution measurements. This integrated approach improves 1-day-ahead ozone forecasts, particularly in areas where local resolution is lacking. The results show that combining these models enhances forecast accuracy and provides better alert systems for exceeding ozone thresholds in Slovenia.

In the study [54], a comparison of the performance of multiple linear regression and multilayer perceptron in predicting the ${\mathrm{SO}}_{2}$ concentration in Tehran’s air is conducted. Different parameters, such as meteorological conditions, urban traffic data, green areas, and time variables, were used for the analysis. The results show that the MLP model achieved better performance in terms of $R^{2}$ and RMSE measures than the MLR. Sensitivity analysis showed that the most important factors affecting the ${\mathrm{SO}}_{2}$ level are the one-day time lag, park index, season, and total park area. The MLP model can support the analysis and air quality management strategies, emphasizing the importance of artificial neural networks in reducing urban pollution.

### 3.5. Other DL Approaches

In [55], recurrent artificial neural networks to estimate ${\mathrm{SO}}_{2}$ and PM_10_ air pollution levels in Sakarya (Turkey) were proposed. The results showed strong correlations and low RMSE values, demonstrating the efficacy of deep learning methods even under challenging conditions. The study also highlighted that PM_10_ levels in Sakarya often exceeded legal limits, particularly during peak industrial periods.

In [56], the authors proposed an aggregated long short-term memory model for air pollution forecasting. Its performance was evaluated against three commonly used machine learning approaches, using MAE, RMSE, and MAPE as assessment metrics. The experiments were conducted with air quality data collected from multiple monitoring sites across Taiwan.

In [57], a radial basis neural network was employed for air quality prediction in Zhengzhou and Shanghai (China). The study explores the network’s ability to model and forecast air quality based on the collected data from these cities.

The study [58] uses an artificial neural network and wavelet ANN to forecast the air pollution index (API) by analyzing its linear and nonlinear relationships with meteorological variables in Xi’an and Lanzhou. The study identifies optimal input variables for forecasting, which include APIs from the previous days and selected meteorological factors. The WANN model, utilizing Bayesian regularization, outperformed the standard ANN model in both cities, demonstrating its ability to accurately predict API values by capturing nonlinear relationships. The findings suggest that WANNs are effective for short-term API forecasting, potentially aiding in environmental management policies.

The research presented in [59] introduces an artificial neural network-based approach using a feed-forward neural network to predict ${\mathrm{NO}}_{x}$ emissions from diesel-powered underground LHD vehicles. The model achieves predictions with less than 15% error, making it an efficient tool for estimating dynamic behavior in complex systems. The accuracy enables its application in estimating environmental impact and planning ventilation systems in mines. This approach offers insights into underground air quality and enhances safety for mining crews.

The paper [60] considers a factor-augmented autoregressive neural network to forecast ${\mathrm{NO}}_{x}$ pollution levels in Madrid, using lagged ${\mathrm{NO}}_{x}$ values, meteorological data, and latent factors. The model outperforms competing methods, providing more accurate predictions of air pollution, which could aid policymakers in improving air quality monitoring and management.

The study [61] develops a combined weight forecasting model for predicting ${\mathrm{NO}}_{2}$ concentration in Beijing using wavelet-transformed data. Three models using the discrete wavelet transform with long short-term memory, gated recurrent unit, and bi-directional long short-term memory are constructed and compared. The proposed model integrates these models through weighted assignment, utilizing their strengths to enhance prediction accuracy. Results indicate that it outperforms individual models, making it a more effective tool for air quality forecasting and governance efforts.

In [62], a graph convolutional temporal sliding long short-term memory model to predict air pollutant concentrations by capturing spatiotemporal correlations is proposed. The model integrates graph convolutional networks for spatial dependency modeling and long short-term memory networks with a temporal sliding strategy for temporal dependency modeling. Applied to the Beijing–Tianjin–Hebei region, it predicts PM_2.5_, PM_10_, O_3_, CO, ${\mathrm{SO}}_{2}$, and ${\mathrm{NO}}_{2}$ levels for 24 h, outperforming state-of-the-art models in accuracy and stability. The approach supports improved air quality management and decision-making.

The research [63] proposes a DL-Air model, which is a hierarchical deep learning model for air quality forecasting. DL-Air consists of three components: an encoder that captures spatial relationships in air quality data, a variant of LSTM that models temporal dependencies and spatiotemporal correlations, and a decoder that transforms latent predictions into actual forecasts. Evaluated on real-world data from Delhi, DL-Air outperformed baseline models, significantly reducing RMSE and MAE and improving $R^{2}$, while maintaining consistent predictive performance across all seasons.

The study [64] implements a transformer-based model, the temporal fusion transformer, trained on observational data from three European countries, achieving high accuracy in four-day forecasts of a daily maximum 8 h of ozone. The model outperforms other machine learning approaches and generalizes well to data from 13 additional European countries not used in training. Analysis of the model’s attention mechanism shows it focuses on key physical variables and the most relevant past ozone values, highlighting its potential as an efficient tool for air quality forecasting across Europe.

In the work [65], a lightweight deep learning model based on sparse attention transformer networks (STN) with encoder–decoder layers, which reduces time complexity while learning long-term dependencies from PM_2.5_ data, is proposed. Experiments on the Beijing and Taizhou datasets demonstrate that the STN model outperforms state-of-the-art methods in both short-term and long-term predictions, achieving high $R^{2}$ and low RMSE and MAE values. The model remains effective for multi-step forecasts up to 48 h, highlighting its robustness and efficiency for air quality prediction.

The paper [66] presents HighAir, a hierarchical graph network (HGN) for air quality forecasting that utilizes an encoder–decoder architecture and considers factors such as weather and land use. The model combines city- and station-level analysis, employing message-passing mechanisms and specialized strategies for exchanging information between levels. Furthermore, edge weights are dynamically adjusted based on wind direction, allowing for a more realistic representation of pollutant dispersion. Tests conducted on data from the Yangtze River Delta (10 cities, 61,500 km^2^) show that HighAir significantly outperforms existing air quality forecasting methods.

The authors of paper [67], propose an air quality forecasting method based on an attention temporal graph convolutional network (AT-GCN), which combines attention, a recurrent GRU network, and graph convolutional mechanisms. The model was tested on air quality, meteorological, and road traffic data from Madrid. Results compared with other methods (LSTM, GRU) showed that the proposed approach better captures complex dependencies and achieves lower prediction errors. Research indicates the significant potential of AT-GCN for urban pollution prediction applications.

In [68], a spatiotemporal graph network model based on GraphSAGE for forecasting ozone concentrations in urban areas is proposed. The model was trained and tested on data from Houston, Texas, USA, considering different forecast horizons (1, 3, and 6 h), the number of time lags, and data from neighboring stations. The results showed significant improvements over the persistence model—by 33.7%, 48.7%, and 57.1%, respectively—and lower errors compared to the existing literature. SHAP analysis revealed that solar radiation becomes more important at longer forecast horizons, and the model correctly identified exceedances of EPA air quality standards.

The research [69] proposes a group-aware graph neural network (GAGNN), which is a hierarchical model for national-scale air quality forecasting. The model constructs a city graph and a city-group graph to capture both spatial dependencies and hidden connections between distant but correlated locations. A differentiable clustering network is introduced to discover inter-city dependencies and a module for encoding inter-group correlations, and a message-passing mechanism is then applied to the graph. Experiments conducted on large datasets from China and the United States show that GAGNN outperforms existing air quality forecasting models.

In [70], a computational framework based on graph neural networks for air quality prediction in complex urban environments is proposed. This model considers both the spatial distance between stations but also contextual factors such as land use and dominant pollutant sources. Additionally, an approach is developed to handle heterogeneous measurement systems, where stations monitor different sets of pollutants. The effectiveness of the solution was validated on a nationwide Spanish air quality dataset, achieving very promising results.

The paper [71] presents a self-supervised hierarchical graph neural network (SSH-GNN) method for air quality forecasting with high spatiotemporal resolution. The model first approximates the air quality distribution across a city based on historical data and contextual factors such as weather and traffic volume. It then uses a hierarchical recurrent graph network to model long-range spatiotemporal correlations between urban regions. Using spatio-temporal self-supervision strategies, SSH-GNN can effectively capture both universal topological and contextual patterns, resulting in significant improvements in forecast accuracy when tested on two real-world datasets.

The paper [72] presents a real-time air pollution forecasting system for five key locations in Delhi, using historical air quality and meteorological data. The authors propose an end-to-end sequential modeling framework to predict concentrations of pollutants such as ${\mathrm{NO}}_{2}$, PM_2.5_, and PM_10_, and to classify their threat levels for the next 24 h. The system effectively handles missing or unreliable data and demonstrates higher performance compared with baseline forecasting methods.

A comprehensive survey of different techniques using deep learning for air pollution forecasting is presented in [16]. The paper reviews various approaches, including artificial neural networks. The study emphasizes the effectiveness of deep learning in improving the accuracy and reliability of air quality predictions, highlighting their ability to model complex, non-linear relationships between meteorological factors, pollutant concentrations, and other influencing variables. It also discusses the challenges of implementing these techniques in real-world air quality forecasting systems, such as data quality and computational cost.

The deep learning methods are summarized in Table 3.

### Discussion

The presented studies confirm the increasing popularity and effectiveness of deep learning methods in forecasting air pollutant concentrations. Artificial neural networks, including multilayer perceptrons, remain a frequent choice due to their flexibility and relatively low computational cost, as shown in [30,31,54]. Several works demonstrate that the performance of such models, measured using statistical indicators such as $R^{2}$, RMSE, MAE, or MAPE, is often satisfactory and applicable in real-world environmental monitoring scenarios. Advanced architectures such as CNN-LSTM [42,43,44] and Bi-LSTM networks [41] are employed to capture both spatial and temporal dependencies in the data, which is particularly important when modeling particulate matter dynamics. Studies using LSTM-based approaches emphasize their strength in modeling long-term temporal dependencies and addressing data sequences with irregular patterns [56]. Some works introduce hybrid or optimized architectures, such as the use of genetic algorithms to enhance ANN performance by selecting the most relevant input variables [31]. The use of CNNs [40] and RNNs [55] further diversifies the methodological landscape, indicating that no single architecture dominates the field, but rather that the choice of method is often tailored to the specific pollutants, data availability, and local conditions.

The most frequently predicted pollutants include PM_2.5_, PM_10_, and ${\mathrm{SO}}_{2}$, reflecting their importance in urban air quality assessment. Several authors emphasize the importance of meteorological and temporal features, such as wind speed, humidity, season, and day lag [54], indicating that the integration of environmental context is crucial for accurate forecasting. The wide range of architectures and pollutants studied demonstrates the adaptability of deep learning approaches, though it also highlights the need for standardized evaluation protocols and benchmark datasets to ensure comparability. Despite the progress, many models remain location-specific and dependent on the quality and resolution of available data.

Importantly, the potential applications of these models extend beyond academic research. High accuracy forecasts of pollutant concentrations can support the development of early warning systems, inform public health advisories, and guide short-term regulatory or traffic-control interventions in urban areas. In the longer term, insights derived from deep learning models may assist policymakers in evaluating the impact of environmental strategies and in optimizing resource allocation for air quality improvement.

A comparative analysis of deep learning methods for air quality forecasting reveals that the suitability of each architecture depends strongly on the characteristics of the available data and the forecasting horizon. CNN-based models are computationally efficient and particularly effective when high-resolution spatial features dominate, for example, in urban-scale pollution mapping. LSTM and Bi-LSTM networks, by contrast, excel at modeling sequential dependencies and capturing long-term temporal patterns, making them useful for predicting daily or seasonal pollutant variations. Transformer-based approaches are emerging as a promising alternative, especially for handling very long input sequences and multimodal datasets, though their practical deployment is still limited by high computational requirements and the need for large annotated datasets. Wavelet-enhanced networks occupy a more specialized niche, but their ability to denoise input signals and capture both time- and frequency-domain information makes them valuable when dealing with irregular or noisy sensor data.

From a practical perspective, CNNs are often applied in short-term urban forecasting tasks where spatial variability is crucial, while LSTMs and hybrid CNN–LSTM models are preferred for continuous monitoring and early warning systems that rely on temporal dynamics. Transformers may find broader adoption in large-scale environmental monitoring platforms that integrate ground sensors, satellite data, and meteorological inputs. Wavelet-based models, though less common, can support scenarios where the reliability of low-cost sensors is limited, as they enhance signal robustness under adverse conditions. Overall, no single architecture dominates; rather, their strengths and weaknesses are complementary. This diversity suggests that future research should emphasize the design of hybrid and ensemble frameworks that combine the efficiency of CNNs, the temporal learning capacity of LSTMs, the scalability of transformers, and the denoising capabilities of wavelets to achieve more accurate and generalizable forecasts across diverse environmental contexts.

Nevertheless, realizing the full potential of these approaches requires addressing several unresolved challenges. One major limitation is the interpretability of deep learning models, which often function as “black boxes”. This can hinder trust in the results, particularly in policy making contexts where transparency is important. Recent work increasingly emphasizes the need for explainable artificial intelligence (AI) methods to address this issue. Additionally, access to high-quality, granular environmental data is not uniform globally. In many regions, deep learning approaches may be hindered by limited data availability or data privacy regulations, raising ethical concerns regarding equitable access to the benefits of AI-driven air quality forecasting.

## 4. Machine Learning Models

In this section, we present methods for forecasting air pollution concentrations based on machine learning. These techniques, including support vector machines (SVM), random forests (RF), linear regression (LR), ensemble learning (EL), or XGBoost, allow for effective modeling of the pollution levels. We will discuss the selected methods, their properties, and workflow.

In the study [73], a system for forecasting NO, ${\mathrm{NO}}_{2}$, $O_{3}$, PM_10_ and PM_2.5_ concentrations for 24 h ahead was developed. The methodology includes characterization, forecasting, and evaluation of the system using meteorological data and information on emission sources. A comparative analysis was conducted between artificial neural networks and the support vector model improved by particle swarm optimization, where SVR-PSO showed the highest forecasting performance. The system operates independently of the season, which confirms its stability and usefulness in air quality management.

In [74], an advanced warning system consisting of two modules is proposed: air quality forecasting and evaluation. The forecasting module uses a model combining data preprocessing and numerical optimization methods, as well as interval forecasting to take into account uncertainty. The evaluation module based on the cloud model enables the transformation of qualitative data into quantitative air quality assessments. The simulations conducted on data from the Chinese city of Dalian confirmed the high efficacy and wide applicability of the proposed system.

Air quality forecasting using machine learning to predict pollutant concentrations in hourly intervals is proposed in [75]. A multi-task learning approach is considered, taking into account meteorological data from previous days. A regularization method is introduced, enforcing similar forecast values in subsequent hours, which improves the model accuracy. The experiments show that the proposed techniques outperform standard regression models and existing regularization methods.

A study presented in [76] explored an ensemble learning method for predicting PM_2.5_ concentrations. This approach utilized climate data and air quality metrics from adjacent cities. The predictive performance was evaluated using datasets containing PM_2.5_ measurements from Beijing, Wuhan, and Shenzhen.

In [77], a machine learning model based on gradient boosting to forecast PM_2.5_ concentrations in Taiwan is introduced. The study included a comparative analysis with data from Taipei and London, revealing similar predictive outcomes for both cities, likely due to their comparable topography. The model’s performance was assessed using the coefficient of determination ($R^{2}$) and RMSE.

The research in [78] developed three machine learning models: multiple linear regression, random forest regression, and the general additive model for accurate forecasting of particulate matter concentrations. The results demonstrated high accuracy across all models, with the GAM model achieving the best predictive performance. Additionally, the study outlines a detailed methodology for predicting PM_10_ variability, offering a framework that can be adapted for different regions.

The study [79] uses the Prophet forecasting model to predict short-term and long-term air pollution in Seoul, including pollutants PM_2.5_, PM_10_, $O_{3}$, ${\mathrm{NO}}_{2}$, ${\mathrm{SO}}_{2}$, and CO. It outperforms similar models with significantly lower prediction errors, especially for PM_2.5_ and PM_10_. PFM’s ability to predict air quality for up to a year shows promise for regions lacking advanced meteorological infrastructure. The study also emphasizes the potential for PFM to guide air quality management in Seoul.

The study [80] addresses the challenge of monitoring benzene ($C_{6}H_{6}$) in urban areas, where sensor deployment is expensive and limited. It proposes an ensemble machine learning approach to estimate $C_{6}H_{6}$ levels by utilizing the relationships between gases. Extensive experiments show that this technique significantly enhances the performance of existing machine learning methods for air pollution monitoring. The approach offers an efficient solution for benzene detection, potentially improving urban air quality monitoring without relying on costly sensor deployments.

The work [81] focuses on improving air quality and reducing human exposure to pollutants, particularly in developed cities in China, where ground-based measurement networks are too dispersed to capture spatial variability in rural and mixed-use areas. The authors utilize high-resolution satellite data, along with meteorological and chemical information from the WRF and CMAQ models, to estimate the vertical columnar ${\mathrm{NO}}_{2}$ density and the distribution of near-surface ${\mathrm{NO}}_{2}$ concentrations. They employ data assimilation methods and probabilistic kernel function models, with Gaussian regression providing the best results. They also analyze atmospheric ${\mathrm{CO}}_{2}$ concentrations using data assimilation and inverse modeling, identifying high-emission areas and interannual variability in fossil fuel emissions in East Asia. The results allow for better monitoring and prediction of long-term changes in ${\mathrm{NO}}_{2}$ and ${\mathrm{CO}}_{2}$ concentrations and provide a baseline for implementing local pollution control measures and environmental policies.

In [82], a method for predicting the AQI is considered. The study includes data from the city of Visakhapatnam (India), covering 12 pollutants and 10 meteorological parameters. Several ML algorithms were tested, with CatBoost performing best, while AdaBoost obtained a lower $R^{2}$ value. The results confirm that machine learning techniques, especially CatBoost, enable highly accurate prediction of urban air quality and can be successfully applied globally.

The study [83] introduces a hybrid PSO–SVM model for short-term air pollutant concentration forecasting. The approach combines influence factor analysis, clustering of input data, and parameter optimisation to enhance predictive accuracy. A case study in Beijing demonstrates that the proposed method outperforms baseline and alternative models, including genetic algorithm-optimised SVM. The results highlight the potential of the hybrid framework as an effective tool for air quality management and early warning systems.

In the paper [84], an air quality forecasting system for Macau using five machine learning algorithms: ANN, RF, XGBoost, SVM, and MLR, to predict PM_2.5_, PM_10_, and CO concentrations over 24- and 48 h time horizons is developed. Measurement data were collected from 2016 to 2021, with 2020 to 2021 used for testing and the previous four years for model training. All models achieved good results for 24 h forecasts and acceptable accuracy for 48 h forecasts, although performance could be improved by feature selection using the SHAP test. The RF and SVM models achieved the best results for both PM_2.5_, PM_10_, and CO over 24- and 48 h forecasts.

The work [85] developed a machine learning model to forecast daily PM_2.5_ concentrations in Shanghai by combining data from the operational numerical system WRF-Chem with ground-level measurements and meteorological conditions. The ML model significantly improved forecast accuracy compared to WRF-Chem alone, achieving 50–100% higher correlation coefficients and reducing the standard deviation. The results show that the use of machine learning can effectively complement chemistry-transport numerical models in air quality forecasting in China. This approach enables more precise pollutant predictions and supports better public health decisions.

A comprehensive literature review describing machine learning methods for modeling air quality may be found in [86]. The machine learning methods are summarized in Table 4.

### Discussion

Machine learning methods have demonstrated considerable potential in the task of air pollution forecasting, particularly due to their ability to model complex, nonlinear relationships between meteorological variables, emission data, and pollutant concentrations. A variety of algorithms have been applied, including support vector machines (SVM), random forests (RF), gradient boosting methods such as XGBoost, multi-task learning, and time series approaches like Prophet. These methods are often combined with data preprocessing and optimization techniques, such as particle swarm optimization or regularization strategies, to improve forecasting accuracy. One of the notable strengths of machine learning models is their adaptability to different input features, such as meteorological data, historical pollutant levels, or even data from nearby regions, as seen in [76]. Moreover, architectures combining prediction with evaluation modules [74] or integrating interval forecasting [75] demonstrate that ML models can go beyond point estimation and support more robust decision making.

Each algorithm presents distinct advantages and limitations. SVM is valued for its robust generalization and interpretability, but can be computationally intensive for large datasets. RF provides high predictive accuracy and relatively fast training while offering feature importance insights, making it easier to interpret. XGBoost delivers state-of-the-art performance on structured data and handles complex nonlinear interactions effectively, although its hyperparameter tuning is more involved. Prophet offers ease of implementation and transparency in time series modeling, yet it may struggle with abrupt changes in pollutant concentrations. These differences illustrate that no single algorithm dominates the field, and the choice often depends on data availability, pollutant type, spatial resolution, and computational resources. Combining multiple ML methods or using ensemble strategies can further enhance forecast reliability and robustness.

The selection of specific machine learning models is often influenced by the characteristics of the target pollutant and the operational context. Particulate matter (PM_2.5_, PM_10_), which exhibits strong temporal and spatial correlations, benefits from models capable of capturing both sequential patterns and feature interactions, such as LSTM-enhanced or CNN–LSTM hybrid architectures. Gaseous pollutants like CO or NO^2^, which show more localized and short-term variability driven by traffic or industrial emissions, are well suited to ensemble methods such as random forest or gradient boosting approaches like XGBoost, which effectively handle heterogeneous features and nonlinear dependencies. Time series–oriented models, including Prophet, are preferred for capturing seasonal patterns or when only coarse historical data is available. Moreover, hybrid or ensemble strategies are commonly adopted in complex urban environments to utilize the complementary strengths of multiple models, enhancing robustness under varying meteorological conditions, sensor coverage, or missing data. This context-specific selection underlines the importance of aligning algorithm choice with pollutant type, data characteristics, and operational requirements to achieve accurate and actionable air quality forecasts.

However, several challenges persist. First, model interpretability remains limited for many complex ML approaches, especially ensemble and black box models, which can hinder their acceptance by environmental agencies or policymakers. Second, generalization across seasons, locations, or cities is often assumed rather than empirically validated. Although [73] claims seasonal independence, such robustness is rarely tested across diverse contexts. Furthermore, while some studies utilize uncertainty quantification or multi-task settings, these techniques are not yet widespread. A significant gap also lies in the comparability of results, as the influence of input feature selection, data quality, and spatial resolution on model performance is often underexplored.

In summary, machine learning methods offer a powerful toolkit for forecasting air pollutants, balancing predictive performance, interpretability, and computational efficiency. Nevertheless, widespread deployment requires more rigorous validation, better explainability, and standardized evaluation practices. Future research should focus on integrating ML models with domain knowledge, heterogeneous sensor networks, and real-time data streams to enhance generalization, resilience, and actionable utility in environmental decision-making.

## 5. Statistical Models

In this section, we present statistical methods used to forecast air pollutant concentrations. Classical approaches, such as regression analysis, autoregressive models (AR), autoregressive models with moving average (ARMA), and their extensions (ARIMA, SARIMA), allow for the analysis and prediction of air quality changes based on historical data. These methods are particularly useful in modeling long-term trends and seasonal patterns of pollutant concentration changes. Although they are often characterized by lower accuracy than modern machine learning and deep learning models, their interpretability and lower computational requirements mean that they are still widely used.

The study [87] focuses on the analysis of PM_2.5_ concentration in Fuzhou (China) using the ARIMA model. The results show that PM_2.5_ shows seasonal fluctuations, with higher values in winter and lower values in summer. Spearman correlation analysis shows that PM_2.5_ is positively correlated with PM_10_, ${\mathrm{SO}}_{2}$, and ${\mathrm{NO}}_{2}$, and is negatively correlated with meteorological parameters. The forecasted values of PM_2.5_ based on historical data suggest a decrease compared to previous years, which may be the result of policy actions taken by Fuzhou authorities.

In [88], the PM_10_ concentrations in Taiyuan (China) were analyzed using a wavelet-based ARMA/ARIMA model. The model’s performance was assessed using multiple error measures. The results showed minimal values for the error metrics, indicating the model’s high accuracy.

The work [89] examines the air quality in Lahore (Pakistan), focusing on the growing pollution issues that conflict with the pursuit of a high quality of life. The study reveals that PM_2.5_ and PM_10_ concentrations exceed the National Environmental Standards (NEQS). Correlation analysis establishes the relationship between particulate matter and other pollutants such as $O_{3}$, NO, and ${\mathrm{SO}}_{2}$, indicating that road traffic plays a significant role in the increase in emissions. A projection of future PM_2.5_ concentrations using the SARIMA model predicts a further rise in particulate matter levels next year. The paper also provides a detailed analysis of air pollutants and their sources, stressing the need for actions to improve air quality in the region.

The research [90] explores the forecasting of monthly PM_10_ concentrations in Erzurum (Turkey) to take measures that minimize the risk of air pollution. The model was developed using the historical data. The data were cleaned from seasonal trends and effects and then decomposed into three levels. ARIMA models were applied to each subseries for forecasting the approximation and detail series. The WT-ARIMA model outperformed the traditional ARIMA in terms of RMSE, $R^{2}$, MAE, and MAPE metrics. The results demonstrate the high accuracy of the model, making it an effective tool for early warning in areas with high air pollution levels.

In [91], a statistical model was developed to predict PM_2.5_ and PM_10_ concentrations, capturing up to 57.0% of the variability in PM_2.5_ and 35.0% in PM_10_. Temperature, wind speed, and wind direction explain 94.0% of the variability in PM_2.5_, while relative humidity is particularly significant for PM_10_. The inclusion of lagged PM concentrations further enhanced the prediction accuracy by 4.0–16.0%. When tested with historical data, the model showed strong correlations with observed PM concentrations and was effective in forecasting levels at additional monitoring sites.

In the work [92], the use of widely accessible LoRa devices and low-cost PM sensors for monitoring PM concentrations is discussed. The study also proposes a short-term forecasting method (up to 2 h) for PM concentrations using ARIMA and VARMA models. Experimental results from data collected by 15 LoRa sensors show a 7.77% improvement in forecast accuracy and a 3.7% increase in the correlation coefficient compared to forecasts made using data from a single sensor.

The paper [93] investigates the application of quantile regression to forecast extreme ${\mathrm{NO}}_{2}$ concentrations in Madrid. Unlike point forecasts, this method allows for predicting the full probability distribution, which allows for better modeling of extreme values. The developed models, using meteorological data, outperform traditional point approaches in terms of accuracy and reliability. The importance of input variables is also analyzed, showing differences between the median and higher quantiles. Furthermore, a method for calculating the probability of exceeding threshold ${\mathrm{NO}}_{2}$ values is presented.

In [94], ARMA and ARIMA models were used to predict daily mean concentrations of $O_{3}$, CO, NO, and ${\mathrm{NO}}_{2}$ in Delhi. Data were subjected to a variance stabilizing transformation, and optimal model parameters were selected based on information criteria and autocorrelation functions. Accuracy was assessed using MAPE, MAE, and RMSE. The results indicate the effectiveness of this method in short-term air quality forecasting.

The study [95] proposes a stepwise multiple linear regression model to forecast PM_10_ and NOx concentrations in Athens and Helsinki, using pollutants and meteorological variables as predictors. Results showed that including Monin–Obukhov length and mixing height improved forecast accuracy, especially for Helsinki. MLR outperformed artificial neural networks in some cases, providing reliable predictions for daily averages and maximum hourly concentrations. The study concluded that MLR models are a useful tool for regulatory air quality forecasting despite limitations, such as the inability to capture rapid meteorological changes.

In the work [96], the ARIMA model to predict atmospheric environmental quality in Hunan Province (China) is proposed. The findings suggest a year-on-year improvement in air quality, with forecasts indicating gradual enhancement. The model proves reliable for predicting air pollutants (PM_2.5_, PM_10_, ${\mathrm{SO}}_{2}$, CO), providing valuable insights for future air quality management in the region.

The statistical methods for air pollutants forecasting are summarized in Table 5.

Statistical models each come with specific assumptions, typical applications, and inherent limitations. ARIMA models assume stationary time series and linear relationships, making them suitable for short-term pollutant forecasts in urban areas, but they struggle with nonlinear interactions and multivariate data. SARIMA extends ARIMA by modeling seasonal patterns, which is beneficial for regions with strong seasonal effects, yet it requires careful parameter tuning and is less adaptable to high-dimensional datasets. Multiple linear regression (MLR) assumes linearity and independence of predictors, providing rapid estimation across multiple features, though it is sensitive to multicollinearity and less effective for capturing complex nonlinear dynamics. Considering these aspects highlights why statistical models remain relevant and useful, especially in low-data or high-interpretability scenarios, despite the increasing popularity of machine learning and deep learning approaches.

### Discussion

Statistical models, particularly those based on autoregressive techniques, have long been applied in air quality forecasting due to their conceptual simplicity, transparency, and interpretability. Techniques such as ARIMA, SARIMA, and generalized additive models (GAM) allow for straightforward modeling of temporal trends and seasonal patterns, as demonstrated in studies like [87,88,89]. These models provide both accurate forecasts of pollutant concentrations and valuable insights into interrelationships among pollutants and environmental variables. Their interpretability is especially advantageous in policy-making contexts, where understanding the influence of meteorological factors, emission sources, and seasonal effects is crucial.

Despite the rise in machine learning and deep learning approaches, statistical models remain relevant in several scenarios. They require relatively low amounts of data and computational resources, making them suitable for regions with limited monitoring infrastructure, such as parts of Africa or Southeast Asia. In heavily polluted regions like China or India, these models are often used to establish baselines and capture dominant seasonal cycles (e.g., winter smog episodes), while in European or North American contexts, they are valued for their transparency, supporting regulatory compliance and communication with stakeholders. Moreover, they serve as robust benchmarks for evaluating more complex models and can be integrated into hybrid frameworks that combine statistical rigor with data-driven enhancements. Their main limitations include difficulties in capturing nonlinear interactions, limited adaptability to high-dimensional or multivariate datasets, and challenges in utilizing spatial information, which are the issues that are particularly pronounced in regions with complex land use patterns (e.g., urban-industrial corridors or coastal zones) where pollutant dispersion is highly variable.

In addition to the aforementioned considerations, statistical models play distinct roles depending on the forecasting horizon and application. For nowcasting tasks, they are valuable for short-term air quality alerts and rapid identification of pollution peaks, especially in urban areas with limited real-time monitoring. For forecasting and prediction, they help capture seasonal cycles, trends, and long-term pollutant dynamics, providing essential baselines against which machine learning or hybrid models can be benchmarked. In particular, their interpretability facilitates communication with policymakers and stakeholders regarding the expected impact of emission control measures.

The choice of numerical schemes and model structures is also critical. For instance, AR, ARMA, and ARIMA are effective for univariate time series with temporal autocorrelation, while SARIMA utilizes seasonal components. GAMs allow flexible nonlinear relationships between predictors and pollutants, and VARMA or wavelet-enhanced ARIMA address multivariate dependencies and nonstationarity. Moreover, downscaling techniques can enhance the spatial resolution of forecasts, allowing statistical models to use localized meteorological or land-use information, which is especially important in regions with complex terrain or heterogeneous emission sources. These methodological choices determine model performance, robustness, and applicability across different geographical and environmental contexts.

Nevertheless, statistical models can be extended and improved in various ways. Approaches such as wavelet-enhanced ARIMA [90] or multivariate structures like VARMA [92] have shown promise in addressing nonstationarity and multivariate dependencies, although these methods are still underutilized compared with machine learning techniques. Furthermore, their strong reliance on temporal autocorrelation means that careful preprocessing is often necessary, especially in heterogeneous urban environments or areas with strong meteorological variability, such as mountainous regions. Despite these challenges, the simplicity and transparency of statistical methods make them an indispensable component of the air quality forecasting toolbox, particularly in low-data or high-interpretability scenarios.

To sum up, statistical methods remain a valuable benchmark for evaluating the performance of more advanced models. They are especially appropriate in data-scarce environments or as part of hybrid systems combining statistical foundations with machine learning enhancements. Future research may benefit from integrating statistical rigor with the flexibility of modern data-driven approaches, thereby improving robustness and interpretability in air quality forecasting across diverse geographical contexts and environmental settings.

## 6. Hybrid Models

In this section, we present hybrid methods that combine different approaches to improve the performance of air pollutant concentration forecasting. A hybrid model in the context of air quality forecasting can be defined as a framework that combines two or more modeling paradigms (e.g., statistical, machine learning, physical, or optimization-based approaches) in order to utilize their complementary strengths while compensating for individual weaknesses [97]. For example, ARIMA models can be used to capture temporal trends, while neural networks or XGBoost algorithms can better handle nonlinearities in the data. Another popular approach is to combine classical regression methods with deep learning models, which allows for a more precise representation of the relationships between meteorological factors and air quality.

### 6.1. Deep Learning + Statistical Methods

The study [98] explores statistical regression and computational intelligence-based models for forecasting hourly ${\mathrm{NO}}_{2}$ concentrations at the Taj Mahal in Agra, with an emphasis on public health-related air quality predictions. The research utilized historical air pollution data, focusing on months when pollution levels peaked. The forecasting models were developed using multiple linear regression and a principal component analysis combined with an artificial neural network method. The proposed model outperformed multiple linear regression, showing higher correlation coefficients and better statistical alignment, and proving to be a reliable tool for predicting air pollution levels at the Taj Mahal.

The paper [99] explores various machine learning techniques for predicting PM_10_ concentrations in Lublin, Poland. The methods assessed include linear regression, *k*-nearest neighbors regression, support vector machine, regression trees, Gaussian process regression, artificial neural network, and long short-term memory networks. Based on the coefficient of determination ($R^{2}$), the experimental analysis found that the artificial neural network provided the most accurate predictions among the methods tested. Further studies on air quality analysis in Poland are available in [100,101].

In [102], a hybrid approach for forecasting PM_2.5_ concentrations is proposed. This method combines a gradient-boosted regression tree with convolutional neural networks and fuzzy *k*-nearest neighbor. The forecasting results were compared with several methods, including multiple linear regression, stacked long short-term memory, bi-directional gated recurrent unit, and gradient-boosted regression tree. Evaluation based on metrics such as root mean square error, mean absolute error, and mean absolute percentage error demonstrated that the proposed method outperforms the others in prediction accuracy.

The work [103] addresses urban air quality forecasting, particularly focusing on the complex temporal patterns that are challenging to predict, especially during extreme events. The authors propose a hybrid model that combines autoregressive integrated moving average (ARIMA) and artificial neural networks to enhance forecast accuracy. The model is applied in Temuco (Chile), where firewood burning is the primary source of PM_10_ pollution during the winter. Meteorological data and PM_10_ measurements are used in the model. Experimental results indicate that the hybrid model outperforms individual models such as ARIMA, artificial neural network, and multilinear regression, successfully forecasting 100.0% of alarm episodes and 80.0% of pre-alarm episodes. This approach demonstrates its potential for application in air quality forecasting across various cities and countries.

In [104], effective methods for forecasting PM_10_ and PM_2.5_ are examined. The study compares various forecasting models, including neural networks (multilayer perceptron, radial basis function, and echo state networks), as well as statistical methods such as the autoregressive model and autoregressive moving average model. The best forecasting results were achieved by combining different types of neural networks.

In [105], ${\mathrm{NO}}_{2}$ concentrations were predicted at 14 measuring stations in the UAE. Statistical and machine learning models were used: ARIMA, SARIMA, LSTM, and NAR-NN in open and closed-loop architectures. Accuracy was assessed using MAPE, and it was found that MAPE strongly correlates with the relative standard deviation of ${\mathrm{NO}}_{2}$ concentrations.

The study [106] compares multilinear regression and multilayer perceptron in predicting ${\mathrm{NO}}_{2}$ concentration in Tehran’s air based on meteorological data, urban traffic, and green areas. The results show that the multilayer perceptron obtains higher forecasting accuracy. Sensitivity analysis indicates that green areas have a key impact on ${\mathrm{NO}}_{2}$ reduction, even surpassing traffic density. A multilayer perceptron is proving to be an effective tool for modeling complex environmental interactions.

The paper [107] proposes a hybrid model combining an artificial neural network and a support vector machine algorithm to improve air quality forecasting accuracy. The method enhances traditional ANN and SVM predictions by using a Taylor expansion-based error correction approach. The models utilize historical air pollution data and meteorological variables to forecast PM_10_ and ${\mathrm{SO}}_{2}$ concentrations in Taiyuan (China). Experimental results using a two-year dataset demonstrate that the hybrid models effectively reduce forecasting errors, offering a promising approach for improving air quality predictions in urban environments.

In [108], an evaluation of three forecasting methods: Elman neural networks, ARIMA models, and a hybrid approach combining both to predict ${\mathrm{SO}}_{2}$ pollution episodes near a coal-fired power station is provided. The hybrid model outperformed the individual methods, achieving the best forecast accuracy. This predictive capability is crucial for power plants, allowing them to take preventive measures to reduce emissions and decrease environmental impact.

The research [109] discusses a long short-term memory network to predict ${\mathrm{NO}}_{x}$ emissions in Pohang (South Korea), using stochastic regression for missing data imputation. The model’s performance is evaluated through the mean absolute scaled error, indicating its ability to outperform a simple naive prediction. The study focuses on optimizing parameters like time windows and learning rates, making it a valuable tool for companies to meet regulatory quotas and reduce costs.

In [110], an evaluation of machine learning models for air pollution forecasting, focusing on support vector regression, ARIMA, and long short-term memory, is considered. Using time series data for PM_10_ and PM_2.5_, the models were tested for a predictive accuracy. Results indicate that SVR and ARIMA are the most effective for forecasting air pollutant concentrations.

In the work [111], an improved air quality index (AQI) prediction model utilizing historical and current meteorological datasets is presented. It evaluates various classifiers on India’s Air Pollution Geocodes Dataset. The decision tree classifier achieved 99.7% accuracy, improving slightly with the random forest classifier. Forecasting was conducted using ARIMA, providing AQI projections for the next 45 days and monthly forecasts for a year. The study highlights how integrating multiple models enhances accuracy, with decision trees and ensemble methods yielding the best results.

### 6.2. Deep Learning + Optimization and Metaheuristics

The work [112] presents a multi-stage forecasting approach for PM_2.5_ concentrations using a novel hybrid model that integrates various machine learning techniques. The proposed method combines wavelet packet decomposition, particle swarm optimization, back propagation neural network, and the AdaBoost algorithm. The results showed that the usage of PSO and AdaBoost significantly improves forecasting accuracy over other methods.

The study [113] explores the forecasting of air pollution, which can assist urban planning for maintaining a sustainable environment and reducing health risks. The study focuses on predicting PM_2.5_ concentration at two air monitoring stations in Kuala Lumpur (Malaysia), using hybrid deep learning models. These models, based on 4 h data, utilize six types of pollutants, meteorological parameters, and data from nearby stations. Particle swarm optimization (PSO)- and sparrow search algorithm (SSA)-optimized LSTM models (PSO-LSTM, SSA-LSTM) were developed to identify the most significant combinations of input data. The selected configurations were then applied to empirical mode decomposition (EEMD) to create models: EEMD-PSO-LSTM and EEMD-SSA-LSTM. These models showed notable improvements in forecast accuracy. Among these, the EEMD-SSA-LSTM model outperformed the others, yielding better RMSE and MAE results at the chosen air monitoring stations. The analysis demonstrated that utilizing data from nearby PM_2.5_ stations significantly enhances prediction accuracy, and SSA was found to be more effective than PSO in optimizing LSTM parameters.

In [114], an adaptive neuro-fuzzy inference system combined with a genetic algorithm to model ${\mathrm{NO}}_{x}$ emissions from a natural gas-fired combined cycle power plant is utilized. The model was trained using emission data, showing high predictive accuracy. The results suggest that the genetic algorithm significantly improves the model’s performance, reducing error and enhancing predictive accuracy. The model demonstrated potential for accurate ${\mathrm{NO}}_{x}$ emissions forecasting, with minimum error values across multiple criteria.

The work [115] introduces a hybrid intelligent model combining long short-term memory and the multi-verse optimization algorithm (MVO) to predict ${\mathrm{NO}}_{2}$ and ${\mathrm{SO}}_{2}$ emissions from a Combined Cycle Power Plant. The LSTM model acts as a forecasting engine, while MVO optimizes its parameters to reduce prediction errors. Tested on real-world data from a power plant in Kerman (Iran), the model outperformed existing benchmark models, demonstrating higher accuracy under different input configurations, including meteorological factors and lagged pollutant values.

In [116], hybrid models were proposed for forecasting daily concentrations of various particulate matter fractions and the Air Quality Index (AQI) in Romania. The methods employed included input variable selection, machine learning, and regression models. Evaluation using measures such as $R^{2}$ and RMSE demonstrated satisfactory results in forecasting PM concentrations.

### 6.3. Ensemble Methods

The work [117] compares the performance of a multilayer perceptron and support vector regression in forecasting NO and ${\mathrm{NO}}_{2}$ concentrations based on meteorological data and historical measurements in Szeged (Hungary). Principal component analysis was used to reduce the dimensionality of the data, and the grid search method was used to optimize the model parameters. The results indicate that nonlinear methods outperform linear methods, especially in the case of NO. For ${\mathrm{NO}}_{2}$, the improvement is noticeable but less pronounced.

In [118], a two-step methodology to forecast tropospheric ozone ($O_{3}$) levels in the Sines region of Portugal is developed. It combined meteorological, air quality, and industrial emissions data to predict $O_{3}$ concentrations up to 24 h in advance. The approach used classification and regression trees to identify key predictors and multilayer perceptron models for forecasting. The results showed high prediction accuracy, offering the potential for real-time health and environmental advisories.

The study in [119] presents a hybrid forecasting model that integrates a neural network with the NARX model and uses automatic feature selection. This approach enhances the accuracy of PM_2.5_ predictions, particularly for peak concentrations, and provides a clearer understanding of the influence of weather and seasonal conditions. The experimental results demonstrate strong model performance, with high values of correlation coefficients for one-hour forecasts. The proposed method offers more reliable air quality forecasting and improves the interpretation of factors contributing to pollution.

The paper [120] investigates hybrid approaches for short-term forecasting of PM_2.5_ and PM_10_ concentrations in major Polish cities. The study utilizes stepwise regression, tree-based algorithms (random forests and XGBoost), and neural networks. The experimental results validate the high accuracy of these forecasting techniques.

In the study [121], a hybrid model combining neural networks is introduced for predicting PM_2.5_ concentrations. The model integrates graph convolutional networks with long short-term memory networks to forecast the spatiotemporal changes in PM_2.5_ concentrations. Compared with other leading methods, the discussed model demonstrated higher forecasting accuracy in terms of various machine learning metrics, including the correlation coefficient $R^{2}$.

The work [122] introduces a two-step hybrid model for forecasting ${\mathrm{NO}}_{2}$ and ${\mathrm{SO}}_{2}$ concentrations in four cities in Central China. The model decomposes pollutant data using complete ensemble empirical mode decomposition (CEEMD), then applies support vector regression (SVR) combined with Cuckoo Search (CS) and Grey Wolf Optimizer (GWO) to predict high- and low-frequency components. The final forecast is obtained by summing both predictions. Evaluation results using MAE, MAPE, and RMSE confirm that the CEEMD-CS-GWO-SVR model outperforms other forecasting approaches in accuracy.

### 6.4. Boosting Models

In [123], the particle swarm optimization and artificial bee colony techniques to forecast ${\mathrm{CO}}_{2}$ emissions in Turkey, using socioeconomic indicators such as energy consumption, population, and the number of vehicles, are described. Models are developed in linear, exponential, and quadratic forms, utilizing ${\mathrm{CO}}_{2}$ historical emissions data. The sum of squared error is used as a fitness function. In [124], a sensor array combined with a multilayer perceptron and long short-term memory model to enhance gas concentration estimation in outdoor environments, addressing issues like temperature, humidity, and cross-sensitivity interference, is used. The experimental results demonstrate improved accuracy in predicting CO, ${\mathrm{NO}}_{x}$, ${\mathrm{NO}}_{2}$, and $C_{6}H_{6}$ concentrations, in terms of $R^{2}$ measure, showcasing the efficacy of the MLP-LSTM model in gas sensing applications.

The study [125] proposes a hybrid air pollution forecasting model integrating stacking-based ensemble learning and deep learning frameworks. The approach combines multiple machine learning models, including gradient-boosted tree regression, support vector machine-based regression, and long short-term memory to enhance predictive accuracy for air quality. Experimental results demonstrate the hybrid model’s advantage over traditional models, highlighting its efficacy in improving air pollution forecasting performance.

### 6.5. Other Hybrid Methods

The research [126] discusses a new hybrid forecasting system for PM_2.5_ concentrations. The system begins with an efficient data analysis method to extract the main features from the PM_2.5_ dataset while minimizing noise influence. The Harris hawk optimization algorithm is then used to tune the extreme learning machine model, which achieves high prediction accuracy. The health impacts and economic costs of pollution are estimated based on the predicted PM_2.5_ levels. Experiments conducted with real data from Beijing, Tianjin, and Shijiazhuang show that the proposed system can support environmental management, emission reduction efforts, and health prevention strategies. It is also applicable to various fields, such as PM_2.5_ health research.

In the study [127], a support vector machine kernel designed for predicting the atmospheric pollutant factor PM_10_ in time series problems is presented. The proposed kernel uses a transformed particle swarm-optimized artificial neural network weight vector within a Bayesian-optimized SVM kernel. Experimental results demonstrate that such a new kernel significantly improves forecasting accuracy compared to conventional ANN and SVM models. The findings suggest that the proposed SVM kernel offers an enhanced forecasting technique for air pollution prediction. The machine learning algorithms were also analyzed in [128].

In [129], a time series modeling to predict the concentrations of major pollutants using the adaptive neuro-fuzzy inference system, which better accounts for nonlinear relationships than traditional models, is proposed. The concentrations of CO, ${\mathrm{SO}}_{2}$, $O_{3}$, and ${\mathrm{NO}}_{2}$ data were used for evaluation. The results show that the coefficients of determination for the proposed model are higher than those obtained for the semi-experimental model. The improved forecasting accuracy can support the development of effective environmental and public health policies, contributing to sustainable development.

The study [130] addresses ${\mathrm{NO}}_{x}$ measurement delays in coal-fired power plants, which lead to issues in ammonia injection and compliance with environmental standards. A hybrid boosting model is proposed to predict ${\mathrm{NO}}_{x}$ levels accurately and compensate for measurement delays, improving ammonia injection for denitrification. The model, tested on actual plant data, outperforms ARIMA in short-term forecasts, with reduced errors. This model is suitable for real-time on-site implementation as a soft sensor for power plants.

In [131], a combination of the random forest algorithm with NASA’s GEOS-CF product to forecast $O_{3}$ and ${\mathrm{NO}}_{2}$ concentrations in southeastern China is discussed. The model provides continuous, spatiotemporal forecasts for up to five days, significantly improving upon the initial GEOS-CF model by reducing errors. It offers a more accurate and comprehensive approach to monitoring air quality, which is crucial for decreasing the harmful effects of these pollutants.

The research [132] proposes a hybrid prediction method combining empirical mode decomposition (EMD), a transformer, and a bidirectional LSTM (Bi-LSTM) network, designed for ultrashort-term forecasts of nonlinear time series. The AQI data is first decomposed into intrinsic mode functions (IMFs), then each IMF is predicted separately using the improved transformer-Bi-LSTM approach, with linear prediction applied to simpler trends. Finally, the predicted IMFs are integrated using Bi-LSTM to produce AQI forecasts, achieving low RMSE, MAE, and MAPE values over a 5 h window. Validation on datasets from multiple other cities demonstrates that the model is both highly accurate and broadly applicable for real-time air quality prediction.

A review of hybrid methods for air quality forecasting is also provided in [133,134,135,136,137,138]. The hybrid methods for air pollutants forecasting are summarized in Table 6.

### Discussion

Hybrid methods used to forecast air pollutant concentrations offer significant advantages over traditional forecasting techniques. They combine the power of different algorithms, such as neural networks, regression, principal component analysis, and optimization techniques, to improve the accuracy and interpretability of the results. Hybrid models, such as ANN with NARX in [119] or PCA with ANN in [98], show higher correlation coefficients and better forecast accuracy compared to single methods. These models are particularly effective in predicting pollution during peak periods, where the quality of forecasts is crucial for protecting public health.

The diversity of hybrid approaches in air pollution forecasting can be organized into four main categories. Deep learning + statistical hybrids (e.g., ARIMA + LSTM) utilize the interpretability of classical models while benefiting from the nonlinear learning capacity of DL architectures. Deep learning + optimization and metaheuristics approaches (e.g., PSO- or MVO-optimized LSTM) focus on parameter tuning and convergence, typically yielding performance gains in predictive accuracy and training efficiency. Ensemble methods combine multiple learners, such as RF, SVM, or ANN, to improve robustness and reduce variance, making them suitable for heterogeneous or noisy datasets. Finally, boosting models like XGBoost or gradient boosting trees have gained popularity due to their strong accuracy in capturing complex nonlinearities, albeit with higher training costs and reduced interpretability. This categorization both clarifies the methodological landscape and also emphasizes the trade-offs researchers face when selecting hybrid approaches.

Hybridization improves forecasting performance primarily because it allows different methods to compensate for each other’s weaknesses. Statistical models provide interpretability and capture seasonality or long-term trends, while machine and deep learning models excel at modeling nonlinearities and high-dimensional feature interactions. Optimization and metaheuristic techniques further refine model parameters, leading to faster convergence and reduced risk of local minima. Ensemble and boosting strategies increase robustness by combining diverse learners, which helps to prevent overfitting and enhances generalization. Such synergies are particularly valuable in scenarios where pollutant dynamics are influenced by multiple interacting factors, such as rapidly changing meteorological conditions, mixed emission sources, or heterogeneous spatial settings. In these contexts, hybrid approaches often outperform single models by delivering more accurate, resilient, and context-sensitive forecasts.

Despite these advantages, there are several challenges associated with the implementation of hybrid methods. First of all, the development of such models requires access to large, high-quality data sets, which can be problematic, especially in countries with limited access to pollution data. In addition, the selection of appropriate algorithms and optimization methods requires a thorough analysis of the specifics of a given region and the type of pollutants. It should be noted that each component of the hybrid model may introduce additional complexities and risks of overfitting, especially when data are limited. Another challenge is the interpretation of results, especially in the context of complex, nonlinear relationships between meteorological variables and pollution levels. Although methods such as ANFIS [129] allow for better capturing of these relationships, there is still a need for further development of tools for visualization and explanation of forecasts that will allow their practical application in air quality management. Additionally, many of the presented models rely on advanced computational techniques that require large computational resources, which may be a barrier to their wider implementation in real-time operational systems. This requires the use of computational technologies that can meet the requirements for both speed and accuracy of forecasts.

Moreover, in the context of air pollution modeling, it is necessary to take into account local and seasonal variability, which is a significant challenge, especially in cities with dynamic atmospheric conditions. Although hybrid models offer advanced approaches, much remains to be done to develop forecasting systems that can take into account local conditions in a scalable manner and are robust to changes in input data.

In general, hybrid models that integrate statistical approaches with machine learning tend to perform better than either approach alone, as they can capture both linear dependencies and complex non-linear patterns. For regions with highly dynamic or heterogeneous environments (e.g., mixed urban–industrial areas or complex topographies), hybrid methods that combine physical or chemical transport models with data-driven learning are often more robust. However, the relative advantage of each type of hybrid depends strongly on data availability and the specific environmental context, which suggests that no single hybrid architecture can be universally recommended.

In summary, hybrid methods are a promising alternative to traditional air quality forecasting models, but their effectiveness largely depends on the quality of data, the appropriate selection of methods and algorithms, and the ability to interpret results in the context of changing atmospheric conditions.

## 7. Other Methods

The study in [139] examines hourly PM_2.5_ concentration forecasting in southwestern Poland using the uEMEP and EMEP4PL models. Forecast accuracy is assessed by comparing the results with data from measurement networks. EMEP4PL, an adaptation of the EMEP MSC-W chemistry transport model for Poland, operates with two nested domains: a European grid (12 km × 12 km) and a more refined Polish grid (4 km × 4 km). The uEMEP model, employing Gaussian modeling principles, further downscales EMEP4PL results using the local fraction approach. Findings reveal that uEMEP forecasts exhibit lower bias and a higher index of agreement than EMEP. Additionally, [140] discusses the use of a random forest algorithm to enhance daily PM_2.5_ concentration estimates produced by the EMEP4PL model in Poland.

The work [141] explores short-term forecasting of PM_10_ concentrations in Krakow using sodar Doppler technology, with a focus on the influence of meteorological conditions on air pollution levels. The proposed approach utilizes the spectrum representing the amplitudes of signals reflected back to the sodar receiver from a single-frequency sound transmission, whose properties vary based on atmospheric conditions. Data mining techniques were applied to predict episodes of high PM_10_ concentrations. In [142], particulate matter concentrations in selected Polish cities, including Wrocław and Poznań, from 2014 to 2016 were analyzed. The analysis revealed that the highest concentrations of PM_1.0_, PM_2.5_, and PM_10_ occurred in 2016 in both cities. The study [143] proposes a method for predicting the number of days with atmospheric visibility in Warsaw, Poland, using meteorological and air quality data. The approach employs the Iman–Conover method to simulate a time series of meteorological and air quality parameters. Statistical validation confirmed that the model accurately predicts the number of days within a month when visibility falls within specific ranges.

In [144], a method for predicting a comprehensive air quality index in Xingtai (China), utilizing the speed of socio-economic development in the forecasting model, is presented. The findings demonstrate that the grey multivariable model employed in the study achieves high predictive accuracy.

The study [145] presents an adaptive model that combines artificial neural networks with wavelet analysis for forecasting daily PM_2.5_ concentrations using remote sensing data and ground observations. The model’s performance was evaluated using the Pearson correlation coefficient, mean absolute percentage error, root mean square error, and mean absolute error. The results demonstrate that the wavelet model outperforms the ANN, showing higher forecast accuracy, better stability, and smaller errors during both the training and validation periods. These findings confirm the efficacy of the WANN model for predicting PM_2.5_ concentrations one day in advance. The daily forecasting is also discussed in [146].

In [147], the PM_10_ concentrations during the winter months (December–January) from 2009 to 2015 in Poland were analyzed. The findings indicate that low wind speeds contribute to higher levels of air pollution. The study used data from three monitoring stations in the region, allowing for the identification of smog episodes (referred to as black smog) and the verification of measurements at a station located near a large incineration plant. The results confirm that wind direction plays a significant role in the movement of pollutants and their local deposition.

The paper [148] focuses on forecasting particulate matter concentrations to aid regulatory planning, emission reduction, and the implementation of early warning systems. The authors introduce the CS-EEMD-BPANN model, which integrates gray correlation analysis (GCA), ensemble empirical mode decomposition (EEMD), cuckoo search algorithm (CS), and backpropagation neural networks (BPANN). A key innovation is the use of GCA to identify potential predictors of particulate matter from other air pollutants (such as CO, ${\mathrm{NO}}_{2}$, $O_{3}$, ${\mathrm{SO}}_{2}$) and meteorological variables (including wind speed and direction, temperature, humidity, and pressure). The model was tested in four Chinese cities with diverse climatic conditions, terrain, and emission sources—Beijing, Shanghai, Guangzhou, and Lanzhou. The analysis found that CO, ${\mathrm{NO}}_{2}$, and ${\mathrm{SO}}_{2}$ are strongly correlated with PM, and their inclusion significantly enhances the model’s performance, confirming the effectiveness of the proposed approach. The analysis of ${\mathrm{CO}}_{2}$ emissions is considered in [149].

The paper [150] describes an upgrade to the NAQFC system aimed at improving PM_2.5_ forecasts. The system now includes real-time emissions from wildfires and windblown dust, reductions in dust emissions from snow- or ice-covered areas, and a shortened life cycle of organic nitrates in the gas chemistry. Testing and evaluations conducted over multiple seasons, compared with the EPA AIRNow monitoring network, showed significant improvements in regional forecasts. For instance, including windblown dust improved forecasts in the Western States, and reducing winter dust emissions resulted in a 52 percent reduction in forecast bias in the north-central United States.

In the study [151], a synoptic meteorological classification was performed to analyze its correlation with particulate matter concentrations at five sampling points in Valladolid (Spain), using a Q-mode clustering technique. The analysis considered 11 meteorological variables and the 500-hPa contour, identifying five synoptic meteorological types, which were further divided into nine synoptic classes. The algorithm was also applied to particulate matter concentrations, resulting in seven pollution levels for each sampling point. The comparison between synoptic types and pollution levels revealed a strong correlation between meteorological conditions and particulate matter concentrations at the 500-hPa contour.

In [152], a dynamic data-driven model for estimating NOx and CO emissions from coal-fired utility boilers, focusing on forecasting emissions, is presented. The model demonstrates higher accuracy compared to static models and can be used in dynamic optimization algorithms to minimize emissions. The results indicate that applying both dynamic and steady-state optimization leads to lower emissions compared to historical plant data.

The study [153] evaluates the impact of assimilating surface ozone and fine aerosol measurements on forecast accuracy. Using the Weather Research and Forecasting–Chemistry model along with the Grid-point Statistical Interpolation tool, an assimilation experiment was conducted over northeastern North America, utilizing hourly data from the AIRNow network. Background error covariance was derived from July 2004 forecasts, and model performance was assessed using forecasts from August and September 2006, both with and without assimilation. The results show that assimilation improves ozone and fine aerosol forecasts, leading to enhanced verification scores for at least 24 h. However, this improvement is partially counterbalanced by rapid model error growth during the initial hours of forecasting.

In [154], forecasting ${\mathrm{CO}}_{2}$ emissions in China’s cement industry using a novel grey prediction model, accounting for uncertainty, is considered. The proposed model, V-GM, demonstrated 97.29% accuracy in simulating historical emissions data. It offers a more flexible approach compared to traditional methods, with improved forecasting capabilities. The model also includes an uncertainty analysis, making it useful for decision makers in achieving reliable, cost-effective predictions for emission decrease and sustainable development.

The work [155] examines the hygroscopic properties of particulate matter (PM), which influence light scattering and absorption. The study investigates the behavior of coarse PM (CPM) and fine PM (PM_2.5_) under varying weather conditions and their effect on visibility. A censored regression model was developed to analyze the relationship between PM concentrations and meteorological factors, which facilitated the calculation of the optical hygroscopic growth factor and hygroscopic mass growth. These metrics were applied to PM_2.5_ field measurements using low-cost sensors in two distinct regions. The findings show that CPM and PM_2.5_ concentrations negatively affect visibility, with relative humidity (RH) significantly modulating these impacts. The modeled hygroscopic growth factors closely matched observed values, especially under haze and mist conditions. By adjusting PM_2.5_ concentrations for RH based on visibility-derived growth factors, the accuracy of low-cost PM sensors was notably improved. This research highlights the importance of considering PM-meteorology interactions in visibility forecasting and sensor calibration.

In [156], a fuzzy combination forecasting system to enhance the accuracy and stability of air pollutant predictions is proposed. The system integrates data preprocessing, fuzzy theory, and a Cuckoo Search optimization algorithm to determine optimal model weights. Maximizing decorrelation and maintaining model diversity improves prediction reliability. Experiments using PM_10_ and PM_2.5_ datasets from three cities demonstrate higher performance in accuracy, stability, and generalization, making the system highly effective for air quality early-warning applications, compared to state-of-the-art methods.

The paper [157] discusses the interpretability of a machine learning model predicting ${\mathrm{NO}}_{2}$ concentration in Madrid. The Shapley Additive Explanations method is used to analyze the impact of individual features on the neural network predictions. Three different interpretation methods are also compared to determine their suitability for air quality data analysis. The results provide deeper insight into the model’s performance and allow for a better understanding of its decisions.

In [158], an extended attention model for sequence-to-sequence recurrent neural networks designed to capture periodic patterns in time series is introduced. When applied to univariate and multivariate time series forecasting, the model demonstrates state-of-the-art performance. The model can be integrated with any RNN architecture, enhancing its ability to handle time series with underlying periodicity, thus improving forecasting accuracy.

The work [159] compares 10 state-of-the-art quantile regression models, focusing on forecasting up to 60 h ahead for forecasting ${\mathrm{NO}}_{2}$ concentration. Instead of using raw quantiles, the study derives distribution parameters, making the approach semi-parametric. Quantile gradient-boosted trees perform best for both expected values and full distributions. However, a simpler quantile *k*-nearest neighbors model combined with linear regression achieves comparable results with lower computational costs. The findings highlight the advantages of probabilistic forecasting in air quality management, aiding authorities in activating traffic restrictions proactively.

The study [160] proposes a predictive model for ${\mathrm{SO}}_{2}$ emissions, aiming to estimate high-emission episodes in advance. Unlike previous studies focusing on mean values, this approach uses quantile curves from additive models to capture the full distribution of pollution levels. A back-fitting algorithm with local polynomial kernel smoothers was applied, with bootstrapping used for hypothesis testing. The model was validated using simulated and real ${\mathrm{SO}}_{2}$ data from a coal-fired power station in northern Spain, demonstrating its efficacy in forecasting pollution incidents.

The study [161] analyzes ${\mathrm{SO}}_{2}$ air concentrations in Krakow (Poland) using data from two monitoring stations. The research identifies trends, seasonal variations, and cyclic patterns, applying a multiplicative time series model. Despite a slight long-term decline, an initial upward trend was observed. The study also examines wind direction effects, finding no significant impact, suggesting local emissions as the dominant pollution source. The methodology, validated through monthly and quarterly forecasts, is adaptable for analyzing other air pollutants in different cities.

Numerical chemical transport models, such as WRF-CHEM [162] and WRF-CMAQ [163], are widely used for simulating atmospheric pollutant dispersion and chemical transformations. These models provide high-resolution spatiotemporal forecasts, supporting both short-term nowcasting of pollution peaks and longer-term scenario analyses. They can be coupled with ground-based, satellite, or UAV measurements to improve forecast accuracy and enable comprehensive environmental assessments.

### Discussion

The methods presented in this section illustrate the diversity of approaches applied to air pollution forecasting beyond conventional statistical, machine learning, or hybrid models. Deterministic chemical transport models, statistical time series methods, and novel machine learning or metaheuristic techniques each offer unique strengths, such as physical interpretability, data efficiency, or the ability to capture complex nonlinear relationships. Their application highlights the importance of tailoring the forecasting approach to the pollutant type, data availability, and spatial-temporal context.

Despite their potential, these approaches also have limitations. Deterministic models require extensive computational resources and high-quality input data, while statistical models may struggle with nonlinearities and transferability to new regions. Advanced ML and hybrid methods often improve predictive performance but can be complex to implement, computationally demanding, and difficult to interpret for policymakers.

Future research should focus on combining the strengths of different approaches, for example, by integrating physical models with adaptive machine learning or by using probabilistic and interpretability-focused techniques. Such integration could enhance forecast robustness, account for uncertainty, and provide actionable insights for regulatory planning, early warning systems, and public health interventions.

Overall, these methods complement mainstream forecasting approaches, demonstrating that diverse, context-sensitive strategies are essential for accurately predicting air pollutant concentrations and supporting effective environmental decision-making.

## 8. Summary, Open Directions, and Future Work

This article presents a comprehensive narrative review of air pollution forecasting methods, including traditional statistical approaches, modern techniques based on machine learning and deep learning, and hybrid models that combine different paradigms to improve prediction accuracy.

Despite significant technological progress, current forecasting methods still face limitations that affect their effectiveness and practical applicability. Many models insufficiently consider dynamic changes in emission sources, such as industrial accidents, wildfires, or traffic surges, and they often rely solely on historical data, limiting adaptability to new environmental conditions. In addition, forecasts frequently lack fine spatial resolution and do not use micro-scale meteorological phenomena. Moreover, few approaches explicitly account for the long-term impacts of climate change on pollutant dispersion and accumulation. Another important challenge remains the limited interpretability of advanced machine learning models, particularly deep neural networks.

Table 7 summarizes the main categories of data-driven forecasting methods reviewed in this paper, highlighting their typical techniques, strengths, and limitations. This synthesis supports the critical comparison of approaches and provides context for the discussion of future research directions.

Open directions and future work

The field of air pollution forecasting is undergoing a rapid transformation due to advances in artificial intelligence, sensing technologies, and computational infrastructure. Among the most promising developments are novel AI architectures. Transformer-based models and attention mechanisms are emerging as powerful tools for capturing long-range temporal dependencies and multi-scale patterns in air quality data. Similarly, graph-based neural architectures allow for explicit representation of spatial relationships between monitoring stations, enabling improved predictions in geographically diverse regions.

Aircraft-based platforms, including drones, are increasingly employed to enhance air quality monitoring. These systems allow for high-resolution measurements in regions with complex terrain, industrial areas, or urban hotspots, providing complementary data that can be integrated with ground-based measurements and numerical models. Utilizing these synergistic approaches can improve the temporal and spatial resolution of pollutant retrieval and enhance forecasting accuracy, particularly in areas where traditional monitoring networks are sparse. Such integration supports more robust and actionable predictions for environmental management and policy decisions [164,165]. Despite these advantages, several open issues remain. Operational costs, regulatory restrictions, and the need for specialized personnel can limit widespread adoption. Additionally, data integration from heterogeneous sources, calibration of sensors, and handling large datasets in real time are ongoing challenges that require further research and development.

Recent large-scale satellite missions have demonstrated the practical integration of remote sensing, numerical modeling, and in situ measurements for comprehensive air quality retrieval and forecasting. For instance, the GEMS mission in Korea [166] and TEMPO in the United States [167] provide hourly observations of key pollutants across wide geographic regions, enabling near-real-time monitoring and policy support. Similarly, NASA missions such as Aura [168] and Sentinel-5P [169] have been extensively used to synergize satellite data with ground-based networks and chemical transport models, improving the spatial and temporal resolution of pollutant estimates. The Suomi National Polar-orbiting Partnership (Suomi NPP) mission, carrying instruments such as VIIRS and CrIS, provides critical observations of aerosols, clouds, and meteorological parameters that support both air quality forecasting and climate research [170]. NISAR, a joint NASA and ISRO mission, is designed to monitor changes in Earth’s surface, including glacial melting, ground movements, and seismic activity [171]; data from this mission can also inform assessments of how environmental dynamics affect air quality. These initiatives exemplify how multi-source data fusion can enhance large-scale environmental assessments, offering valuable insights for air quality management. Future work may utilize these datasets in combination with local low-cost sensors and machine learning algorithms to develop more accurate, scalable, and robust forecasting frameworks.

Numerical chemical transport models, such as WRF-CHEM [162] and WRF-CMAQ [163], are widely used for simulating atmospheric pollutant dispersion and chemical transformations. These models provide high-resolution spatiotemporal forecasts, supporting both short-term nowcasting of pollution peaks and longer-term scenario analyses. They can be coupled with ground-based, satellite, or UAV measurements to improve forecast accuracy and enable comprehensive environmental assessments. For example, in [172], satellite-derived ${\mathrm{NO}}_{2}$ columns over southern China were combined with meteorological outputs from the WRF model to produce high-resolution retrievals. The study demonstrated that integrating WRF data improves spatial accuracy, particularly in regions with complex terrain and large variability, and enables effective validation with ground-based MAX-DOAS measurements. Similarly, in [173], WRF-Chem simulations over southeastern Brazil were evaluated against satellite and ground-based observations for PM_2.5_, NOx, CO, $O_{3}$, and aerosol optical depth (AOD). The work highlighted that combining numerical modeling, remote sensing, and ground measurements allows accurate reproduction of pollutant patterns, captures the transport of urban emissions to inland areas, and underscores the value of integrated approaches for regional air quality assessment. Moreover, in a real-time forecasting context, WRF-Chem was used to support flight planning during the NOAA AEROMMA and NASA STAQS 2023 field campaigns [174]. The forecasting system provided 72 h predictions of atmospheric composition, aerosols, and clouds over domains centered on Chicago and New York City. Evaluation against lidar and surface observations showed that while forecast skill decreased with lead time, the system successfully captured the influence of transported pollution, including wildfire smoke from Canada, and identified limitations related to local meteorological simulations. This demonstrates the practical utility of WRF-Chem for operational forecasting and campaign planning, complementing the long-term modeling and retrieval studies discussed above. Recent studies in Southeast Asia have further illustrated the power of combining WRF-Chem with advanced satellite observations. For instance, the launch of the geostationary UV-VIS spectrometer GEMS in 2020 and NASA’s ASIA-AQ mission in early 2024 enabled detailed monitoring of ${\mathrm{NO}}_{2}$ over densely populated regions, including the Seoul Metropolitan Area, Manila, Taiwan, South Korea, and Thailand [175]. High-resolution WRF-Chem simulations (4 km grid) revealed strong sensitivity of ${\mathrm{NO}}_{2}$ columns to model resolution, captured local enhancements not resolved at coarser scales, and highlighted biases in global emission inventories (e.g., EDGAR v5). Integration with in situ airborne measurements further validated model outputs, demonstrating the potential of synergizing WRF-Chem with satellite and field campaign data for accurate, high-resolution air quality assessment and emissions adjustment in rapidly evolving environments.

Beyond technological advancements, the accessibility and openness of air quality data play a crucial role in enabling informed decision-making, fostering citizen engagement, and supporting smart city initiatives. Platforms such as OpenAQ provide raw air quality measurements that can be analyzed locally and globally, yet challenges remain regarding data quality, interoperability, and standardization across cities. Citizen science initiatives, like “Smell Pittsburgh” [176] and “Participatory Action for Citizens’ Engagement” [177], demonstrate how community involvement in data collection and analysis can improve understanding of local air pollution issues and drive effective interventions. Future efforts combining low-cost sensors, satellite data, numerical models, and active public engagement have the potential to create more robust, equitable, and actionable air quality monitoring systems [178,179,180].

Another important direction is the use of federated and privacy-preserving learning, which allows models to be trained collaboratively across multiple institutions or regions without sharing raw data. This approach can address legal and ethical concerns related to privacy, regulatory restrictions, and data sovereignty. In parallel, there is growing interest in multi-modal and multi-fidelity data fusion. By combining ground-based sensor measurements, satellite imagery, chemical transport model outputs, meteorological forecasts, and mobility data, researchers can construct richer and more resilient forecasting frameworks.

Interpretability remains a major challenge in the deployment of deep learning models. Many state-of-the-art systems still operate as “black boxes”, which can limit their acceptance by environmental agencies and policymakers. Alongside interpretability, there is a push toward real-time and edge computing solutions. Deploying forecasting algorithms directly on IoT devices or edge servers reduces latency and dependence on centralized infrastructure, enabling on-site air quality warnings in smart city applications.

Climate change introduces additional complexity to air pollution forecasting. Shifts in atmospheric circulation, the increasing frequency of wildfires, and the occurrence of heatwaves are altering pollution baselines and generating extreme events that existing models may not capture effectively. Utilizing climate-related variables in forecasting frameworks could improve resilience against these emerging challenges. Finally, the integration of forecasting systems into policy making and public health decision support tools remains an open problem. Models that produce actionable alerts, guide traffic or industrial restrictions, and inform urban planning can transform air quality forecasting from an academic exercise into a cornerstone of environmental governance. Key future research directions are as follows:

- Federated and privacy-preserving learning for multi-region, data-sensitive applications;
- Multi-modal data fusion integrating sensors, satellites, chemical transport models, and mobility data;
- Edge computing for real-time forecasting and on-site air quality alerts;
- Explainable AI to improve adoption by policymakers and environmental agencies;
- Adaptive hybrid models capable of responding to dynamic atmospheric, emission, and socio-economic changes;
- Integration of forecasting systems into public health and urban planning decision support tools;
- Development of standardized benchmarks and open datasets for reproducible evaluation.

The progress of the field critically depends on stronger benchmarking practices, as the current lack of standardized datasets, common evaluation metrics, and open repositories makes objective model comparison and reproducibility challenging. Many studies still rely on proprietary or locally collected data, which hinders cross-study evaluation and slows the adoption of best practices. Establishing open, curated benchmark datasets covering diverse regions and pollution scenarios, together with harmonized evaluation protocols, would facilitate fair model comparison, accelerate methodological innovation, and strengthen transparency. Achieving these goals will require interdisciplinary collaboration between environmental scientists, computer scientists, and policymakers, ensuring that advances in forecasting methods are both scientifically rigorous and practically applicable in real-world air quality management systems.

Future research should focus on increasing the transparency of models and developing methods that will allow for a better understanding of the impact of individual factors on air quality. It will be important to develop more adaptive algorithms that automatically adjust to new conditions, such as the introduction of emission regulations or changes in the transport structure. It will also be crucial to develop short-term forecasts that will enable a faster response to smog episodes and warn residents about threats. Moreover, further improvement of the models by integrating different data sources and developing hybrid forecasting methods should be realized. It will be necessary to develop more advanced methods of air quality forecasting that take into account variable atmospheric conditions, such as wind speed, wind direction, humidity, and pressure. These factors have a significant impact on the spread of pollutants, and their accurate modeling can significantly improve the precision of forecasts. Integrating meteorological data will allow for better prediction of sudden increases in pollution and their local effects. The development of such methods will help to prevent smog episodes more effectively and better plan protective measures. Therefore, future research should focus on creating models that take into account both historical data and dynamic changes in atmospheric conditions.

## Figures and Tables

**Table 1:** Selected air quality indices adopted in different countries and regions.

Index	Country/Region	Main Pollutants Considered	Scale	Remarks
AQI	USA, China, India, others	PM_2.5_, PM_10_, O_3_, CO, NO_2_, SO_2_	0–500	Widely used worldwide; health categories from “Good” to “Hazardous”
API	Malaysia (legacy), Hong Kong (before 2013)	PM_10_, SO_2_, NO_2_, O_3_, CO	0–500	Replaced by AQHI in Hong Kong in 2013
AQHI	Canada, Hong Kong (since 2013)	PM_2.5_, O_3_, NO_2_	1–10+	Focused on health risk communication
CAQI	European Union (EEA)	PM_2.5_, PM_10_, NO_2_, O_3_, SO_2_	0–100	Used in Copernicus/EEA air quality maps
DAQI	United Kingdom	PM_2.5_, PM_10_, O_3_, NO_2_, SO_2_	1–10	Public information, combined with health advice
CAI	South Korea	PM_2.5_, PM_10_, O_3_, CO, NO_2_, SO_2_	0–500	Includes both current and forecasted conditions
PSI	Singapore	PM_2.5_, PM_10_, O_3_, CO, NO_2_, SO_2_	0–500	Based on US AQI but adapted to local standards
IAQI	China	Each pollutant separately (PM, gases)	0–500	Used as components of overall AQI

**Table 2:** Advanced sensing techniques for air pollutants forecasting.

Reference	Air Pollutants	Country/Region	Sensors/Techniques
Zafra et al. [17]	PM_10_	Europe/Spain	LoRaWAN
Apostolopoulos et al. [18]	CO, NO, ${\mathrm{NO}}_{2}$, $O_{3}$, PM_2.5_	Europe/Greece	ENSENSIA sensors
Pochwała et al. [19]	CO, ${\mathrm{NO}}_{2}$, PM_10_, PM_2.5_	Europe/Poland	utilizing drones
Jaron et al. [20]	$C_{6}H_{6}$, HCHO, ${\mathrm{SO}}_{2}$, PM_1_, PM_10_, PM_2.5_	Europe/Poland	drones
Johnson et al. [21]	${\mathrm{NO}}_{2}$, PM_10_, PM_2.5_	Europe/England	Enviro-IoT sensors system
Moskal et al. [22]	CO, ${\mathrm{CO}}_{2}$, $H_{2}S$, ${\mathrm{NO}}_{2}$, $O_{3}$, ${\mathrm{SO}}_{2}$	Europe/Germany	ESP32 boards + wireless communication protocol ESP-NOW
De Vito et al. [23]	PM_2.5_	Europe/Norway	Low Cost Air Quality Monitoring System
Kumar et al. [24]	PM_2.5_	Asia/India	SPS30, PMS7003, HPMA115C0-004 sensors
Ken et al. [25]	CO	Asia/India	TinyML + Arduino Nano BLE Sense
Yadav et al. [26]	PM_2.5_	Asia/India	low-cost particulate matter sensors
Xu et al. [27]	PM_2.5_	Asia/Hong Kong	vehicle-mounted sensors
Wang et al. [28]	CO, ${\mathrm{NO}}_{2}$, $O_{3}$, ${\mathrm{SO}}_{2}$, PM_10_, PM_2.5_	Asia/China	low-cost, multi-parameter air quality monitoring system
Medina et al. [29]	CO, ${\mathrm{NO}}_{2}$, $O_{3}$	Sth. America/Colombia	low-cost air quality monitoring platform + drones + LoRA + PTECA

**Table 3:** Deep learning methods for air pollutants forecasting.

Reference	Air Pollutants	Methods
Ahmadi et al. [30]	${\mathrm{CO}}_{2}$	
Cho et al. [31]	${\mathrm{CO}}_{2}$, PM_10_, PM_2.5_	
Sohn et al. [32]	${\mathrm{CH}}_{4}$, CO, NO, ${\mathrm{NO}}_{2}$, $O_{3}$, ${\mathrm{SO}}_{2}$	
Liang et al. [33]	PM_2.5_	
He et al. [34]	PM_2.5_	
Guo et al. [35]	PM_10_, PM_2.5_	ANN
Guo et al. [36]	PM_2.5_	
Gogikar et al. [37]	PM_10_, PM_2.5_	
Gallo et al. [38]	${\mathrm{CO}}_{2}$	
Ramentol et al. [39]	${\mathrm{NO}}_{2}$	embeddings + ANN
Chae et al. [40]	PM_10_, PM_2.5_	CNN
Du et al. [41]	PM_2.5_	CNN + Bi-LSTM
Bekkar et al. [42]	PM	
Gilik et al. [43]	${\mathrm{NO}}_{2}$, ${\mathrm{NO}}_{x}$, $O_{3}$, PM_10_, ${\mathrm{SO}}_{2}$	
Pak et al. [44]	PM_2.5_	CNN-LSTM
Sharma et al. [45]	TSP	
Li et al. [46]	PM_2.5_	
Liu et al. [47]	PM_2.5_	attention + LSTM
Dairi et al. [48]	n/a	attention + VAE
Mengara et al. [49]	PM_10_, PM_2.5_	attention + Bi-LSTM
Liu et al. [50]	PM_2.5_	attention + RNN
Elbaz et al. [51]	PM_2.5_	attention + CNN/GRU
Abdullah et al. [52]	PM_10_	MLP, RBF
Gradivsar et al. [53]	$O_{3}$	MLP
Shams et al. [54]	${\mathrm{SO}}_{2}$	MLP
Kurnaz et al. [55]	PM_10_, ${\mathrm{SO}}_{2}$	RNN
Chang et al. [56]	PM_2.5_	LSTM
Song et al. [57]	AQI	RBN
Guo et al. [58]	API	ANN, WANN
Banasiewicz et al. [59]	${\mathrm{NO}}_{x}$	FFNN
Fernandez et al. [60]	${\mathrm{NO}}_{x}$	FAA-NN
Liu et al. [61]	${\mathrm{NO}}_{2}$	DWT + LSTM; GRU; Bi-LSTM
Mao et al. [62]	CO, ${\mathrm{NO}}_{2}$, $O_{3}$, PM_10_, PM_2.5_, ${\mathrm{SO}}_{2}$	GCN, LSTM
Abirami et al. [63]	CO, ${\mathrm{NO}}_{2}$, ${\mathrm{NH}}_{3}$, $O_{3}$, Pb, PM_10_, PM_2.5_, ${\mathrm{SO}}_{2}$	hierarchical DL
Hickman et al. [64]	$O_{3}$	transformer
Zhang & Zhang [65]	PM_2.5_	sparse attention transformer network
Xu et al. [66]	AQI	HGN
Iskandaryan et al. [67]	${\mathrm{NO}}_{2}$	AT-GCN
Santos et al. [68]	$O_{3}$	spatiotemporal graph network
Chen et al. [69]	AQI	GAGNN
Terroso-Saenz et al. [70]	$C_{6}H_{6}$, CO, NO, ${\mathrm{NO}}_{2}$, ${\mathrm{NO}}_{x}$, $O_{3}$, PM_10_, PM_2.5_, ${\mathrm{SO}}_{2}$	GNN
Han et al. [71]	AQI, CO, ${\mathrm{NO}}_{2}$, $O_{3}$, PM_10_, PM_2.5_, ${\mathrm{SO}}_{2}$	SSH-GNN
Dua et al. [72]	${\mathrm{NO}}_{2}$, PM_10_, PM_2.5_	sequential modeling

**Table 4:** Machine learning methods for air pollutants forecasting.

Reference	Air Pollutants	Methods
Yu et al. [76]	PM_2.5_	EL
Kaur et al. [80]	$C_{6}H_{6}$	EL
Murillo et al. [73]	NO, ${\mathrm{NO}}_{2}$, $O_{3}$, PM_10_, PM_2.5_	PSO, SVM
Wang et al. [74]	CO, ${\mathrm{NO}}_{2}$, O3, PM_10_, PM_2.5_, ${\mathrm{SO}}_{2}$	NOM
Zhu et al. [75]	$O_{3}$, PM_2.5_	MTL
Lee et al. [77]	PM_2.5_	GB
Eddine et al. [78]	PM	GAM, LR, RFR
Shen et al. [79]	CO, ${\mathrm{NO}}_{2}$, $O_{3}$, PM_10_, PM_2.5_, ${\mathrm{SO}}_{2}$	Prophet
Hugo [81]	${\mathrm{CO}}_{2}$, ${\mathrm{NO}}_{2}$	n/a
Ravindiran et al. [82]	AQI	AdaBoost, CatBoost
Chen et al. [83]	CO, ${\mathrm{NO}}_{2}$, $O_{3}$, PM_10_, PM_2.5_, ${\mathrm{SO}}_{2}$	PSO-SVM
Lei et al. [84]	PM_2.5_, PM_10_, and CO	ANN, RF, XGBoost, SVM, and MLR
Ma et al. [85]	PM_2.5_	XGBoost

**Table 5:** Statistical methods for air pollutants forecasting.

Reference	Air Pollutants	Methods
Zhang et al. [87]	PM_2.5_	ARIMA
Gao et al. [96]	CO, PM_10_, PM_2.5_, ${\mathrm{SO}}_{2}$	ARIMA
Zhang et al. [88]	PM_10_	ARMA, ARIMA
Kumar et al. [94]	CO, NO, ${\mathrm{NO}}_{2}$, $O_{3}$	ARMA, ARIMA
Yun et al. [92]	PM	ARIMA, VARMA
Bhatti et al. [89]	NO, $O_{3}$, PM_10_, PM_2.5_, ${\mathrm{SO}}_{2}$	SARIMA
Aladaug et al. [90]	PM_10_	WT-ARIMA
Afrin et al. [91]	PM_10_, PM_2.5_	statistics
Aznarte et al. [93]	${\mathrm{NO}}_{2}$	QR
Vlachogianni et al. [95]	${\mathrm{NO}}_{x}$, PM_10_	MLR

**Table 6:** Hybrid methods for air pollutants forecasting.

Reference	Air Pollutants	Methods
Mishra et al. [98]	${\mathrm{NO}}_{2}$	MLR, PCA, PCA + ANN
Kujawska et al. [99]	PM_10_	*k*-NNR, ANN, GPR, LR, LSTM, RT, SVM
Usha et al. [102]	PM_2.5_	GBTR + CNN + fuzzy *k*-NN
Diaz et al. [103]	PM_10_	ARIMA + ANN
Neto et al. [104]	PM_10_, PM_2.5_	ESN + AR, ARMA; MLP, RBF
Al et al. [105]	${\mathrm{NO}}_{2}$	ARIMA, LSTM, NAR-NN, SARIMA
Shams et al. [106]	${\mathrm{NO}}_{2}$	MLP, MLR
Wang et al. [107]	PM_10_, ${\mathrm{SO}}_{2}$	ANN + SVM
Sanchez et al. [108]	${\mathrm{SO}}_{2}$	ARIMA, ENN
Lee et al. [109]	${\mathrm{NO}}_{x}$	LSTM + SR
Dobrea et al. [110]	PM_10_, PM_2.5_	ARIMA, LSTM, SVR
Sharma et al. [111]	AQI	ARIMA, DT, RF
Liu et al. [112]	PM_2.5_	AdaBoost, ANN, PSO, WPD
Ahmed et al. [113]	PM_2.5_	PSO- and SSA-optimized LSTM
Dirik et al. [114]	${\mathrm{NO}}_{x}$	ANFIS + GA
Heydari et al. [115]	${\mathrm{NO}}_{2}$, ${\mathrm{SO}}_{2}$	LSTM + MVO
Udristioiu et al. [116]	PM	input variable selection, RM
Juhos et al. [117]	NO, ${\mathrm{NO}}_{2}$	MLP, SVR
Durao et al. [118]	O_3_	MLP, RT
Gu et al. [119]	PM_2.5_	ANN + NARX
Czernecki et al. [120]	PM_10_, PM_2.5_	ANN, RM, RF, XGBoost
Qi et al. [121]	PM_2.5_	GCN + LSTM
Zhu et al. [122]	${\mathrm{NO}}_{2}$, ${\mathrm{SO}}_{2}$	complete ensemble empirical mode decomposition, SVR + Cuckoo Search and Grey Wolf Optimizer
Ozceylan et al. [123]	${\mathrm{CO}}_{2}$	ABC, PSO
Chang et al. [125]	PM_10_, PM_2.5_	GBTR, LSTM, SVM
Du et al. [126]	PM_2.5_	extreme learning machine model
Kouziokas et al. [127]	PM_10_	new kernel for SVM
Zeinalnezhad et al. [129]	CO, ${\mathrm{NO}}_{2}$, O_3_, ${\mathrm{SO}}_{2}$	ANFIS
Lyu et al. [130]	${\mathrm{NO}}_{x}$	boosting model
Li et al. [131]	${\mathrm{NO}}_{2}$, $O_{3}$	RF + GEOS-CF
Dong et al. [132]	AQI	EMD + transformer-Bi-LSTM
Ng et al. [124]	$C_{6}H_{6}$, CO, ${\mathrm{NO}}_{x}$, ${\mathrm{NO}}_{2}$	sensor array + MLP, LSTM
Harishkumar et al. [128]	PM	DT, GBR, MLP, RF

**Table 7:** Comparison of forecasting method categories with selected algorithms/methods.

Category	Typical Methods	Strengths	Limitations
Deep Learning	Autoencoders, ANN, MLP, CNN, LSTM, Bi-LSTM, GRU, RNN, GCN, RBF, Transformer-based models	Captures complex non-linear spatiotemporal patterns; high predictive accuracy; adaptable to multi-modal data; automated feature extraction; supports transfer learning	“Black box” nature; requires large datasets and computational resources; prone to overfitting; location-specific tuning often needed
Machine Learning	SVM, RF, XGBoost, GAM, Prophet, *k*-NN, DT, GB	Handles nonlinearities; moderate interpretability; works with smaller datasets than DL; flexible for various features	Requires manual feature engineering; performance depends on data quality; may struggle with dynamic spatiotemporal patterns; limited generalization across regions
Statistical	AR, ARMA, ARIMA, MLR, SARIMA, QR, VARMA	Simple and interpretable; low computational cost; well-suited for stationary or seasonal patterns; provides uncertainty estimates	Limited to linear relationships; poor spatial modeling; assumes stationarity in many cases
Hybrid	ARIMA+ANN, CNN+LSTM, GCN+LSTM, PSO+LSTM, ANFIS, SSA+LSTM	Combines strengths of multiple paradigms; models physical processes and nonlinear dynamics; integrates heterogeneous data sources	High complexity; requires expertise in multiple paradigms; may inherit limitations of components; higher risk of overfitting

## Data Availability

No new data were created or analyzed in this study. Data sharing is not applicable to this article.

## Appendix A

### Appendix A.1. Abbreviations of Methods Used for Forecasting

The abbreviations of methods cited in this survey are listed in Table A1.

**Table A1:** Abbreviations of methods used for forecasting.

Abbreviation	Method
ABC	artificial bee colony
AI	artificial intelligence
ANFIS	adaptive neuro-fuzzy inference system
ANN	artificial neural network
AOD	aerosol optical depth
API	Air Pollution Index
AR	autoregressive model
ARIMA	autoregressive integrated moving average
ARMA	autoregressive moving average
AT-GCN	attention temporal graph convolutional network
AQI	Air Quality Index
AQHI	Air Quality Health Index
Bi-GRU	Bi-directional gated recurrent unit
Bi-LSTM	Bi-directional long short-term memory
CAI	Comprehensive Air-quality Index
CAQI	Common Air Quality Index
CMAQ	Community Multiscale Air Quality
CNN	convolutional neural network
DAQI	Daily Air Quality Index
DL	deep learning model
DT	decision tree
DWT	discrete wavelet transform
EEA	European Environment Agency
EL	ensemble learning
ELM	extreme learning machine
EMD	empirical mode decomposition
ENN	Elman neural network
ESN	echo state networks
FAA-NN	factor-augmented autoregressive neural networks
FFNN	feed-forward neural network
GA	genetic algorithm
GAGNN	group-aware graph neural network
GAM	general additive model
GB	gradient-boosting-based machine learning
GBR	gradient boosting regression
GBTR	gradient boosted tree regression
GCN	graph convolutional network
GNN	graph neural network
GPR	Gaussian process regression
GRU	gated recurrent unit
HGN	hierarchical graph network
IAQI	Individual Air Quality Index
IoT	Internet of Things
IVS	input variable selection
*k*-NN	*k*-nearest neighbors
*k*-NNR	*k*-nearest neighbors regression
LR	linear regression
LSTM	long short-term memory
MVO	multi-verse optimization algorithm
MLP	multilayer perceptron
MLR	multiple linear regression
MTL	multi-task learning
NAR-NN	nonlinear autoregressive neural network
NARX	nonlinear autoregressive network with exogenous inputs
NOM	numerical optimization methods
PCA	principal component analysis
PSI	Pollutant Standards Index
PSO	particle swarm optimization
RBF	radial basis function
RBN	radial basis network
RF	random forest
RFR	random forest regression
RM	regression models
RNN	recurrent neural network
RT	regression trees
SARIMA	seasonal ARIMA
SR	stochastic regression
SSA	sparrow search algorithm
SSH-GNN	self-supervised hierarchical graph neural network
STN	sparse attention transformer network
SVM	support vector machine
SVR	support vector regression
TSP	total suspended particle
UAV	unmanned aerial vehicle
QC	quantile curves
QR	quantile regression
VAE	variational autoencoder
VARMA	vector ARMA
WPD	wavelet packet decomposition
WT-ARIMA	wavelet transform ARIMA
WANN	wavelet ANN
WRF	Weather Research and Forecasting
WTNN	wavelet transform neural network

### Appendix A.2. Error Measures

Let $x_{i}$ be the actual value, $y_{i}$ is a predicted value and *n* is the number of observations. The $\bar{y}$ denotes the mean value of *y*, and the σ is the standard deviation. The error measures used for forecasting evaluation are listed in Table A2.

**Table A2:** Error measures (with abbreviations) used for forecasting evaluation.

Abbreviation	Error Measure	Equation
ρ	Spearman correlation	(A1)
AAD	absolute average deviation	(A2)
ARV	average relative variance	(A3)
CE	cross entropy	(A4)
DA	direction accuracy	(A5)
$E^{2}$	coefficient of efficiency	(A6)
EM	error mean	(A7)
IA	index of agreement	(A8)
MAE	mean absolute error	(A9)
MAPE	mean absolute percentage error	(A10)
MASE	mean absolute scaled error	(A11)
MFB	mean fractional bias	(A12)
NAE	normalized absolute error	(A13)
NMSE	normalized mean square error	(A14)
NRMSE	normalized root mean square error	(A15)
PA	prediction accuracy	(A16)
R	Pearson correlation coefficient	(A17)
$R^{2}$	coefficient of determination	(A18)
RE	relative error	(A19)
RMS	root mean square	(A20)
RMSE	root mean square error	(A21)
SEP	standard error of prediction	(A22)
SMSE	standardized mean squared error	(A23)
SOS	sum-of-squares	(A24)
SSE	sum square error	(A25)

(A1) $$\rho =1-\frac{6\sum_{i=1}^{n}d_{i}^{2}}{n(n^{2}-1)}$$

where $d_{i}=R\left(x_{i}\right)-R\left(y_{i}\right)$ is a rank difference for each pair $\left(x_{i},y_{i}\right)$, and $R(x_{i})$, $R(y_{i})$ – ranks of appropriate values.

(A2) $$AAD=\frac{\sum_{i=1}^{n}\left(\frac{x_{t}-y_{t}}{x_{t}}\right)}{n}\cdot 100\%$$

(A3) $$ARV=1.0-E^{2}$$

(A4) $$CE=-\sum_{i=1}^{n}x_{i}\ln\left(\frac{y_{i}}{x_{i}}\right)$$

(A5) $$DA=\frac{1}{n}\sum_{i=1}^{n}w_{i},\;w_{i}=\left\{\begin{array}{cc} 1, & \mathrm{if}\;\left(y_{i+1}-y_{i}\right)\left(x_{i+1}-y_{i}\right)>0\\ 0, & \mathrm{otherwise} \end{array}\right.$$

(A6) $$E^{2}=1.0-\frac{\sum_{i=1}^{n}{\left|x_{i}-y_{i}\right|}^{2}}{\sum_{i=1}^{n}{\left|x_{i}-\bar{y}\right|}^{2}}$$

(A7) $$EM=\frac{1}{n}\sum_{i=1}^{n}\left(y_{i}-x_{i}\right)$$

(A8) $$\mathrm{IA}=1-\frac{\sum_{i=1}^{n}{\left(x_{i}-y_{i}\right)}^{2}}{\sum_{i=1}^{n}\left(\right|x_{i}-\bar{y}\left|+\right|y_{i}-\bar{y}{\left|\right)}^{2}}$$

(A9) $$MAE=\frac{1}{n}\sum_{i=1}^{n}\left|\frac{y_{i}-x_{i}}{n}\right|$$

(A10) $$MAPE=100\frac{1}{n}\sum_{i=1}^{n}\left|\frac{x_{i}-y_{i}}{x_{i}}\right|$$

(A11) $$MASE=\frac{\frac{1}{n}\sum_{i=1}^{n}\left|x_{i}-y_{i}\right|}{\frac{1}{n-1}\sum_{j=1}^{n-1}\left|x_{j}-x_{j-1}\right|}$$

(A12) $$MFB=\frac{1}{n}\sum_{i=1}^{n}\frac{x_{i}-y_{i}}{\frac{1}{2}\left(x_{i}+y_{i}\right)}$$

(A13) $$NAE=\frac{\sum_{i=1}^{n}\left|x_{i}-y_{i}\right|}{\sum_{i=1}^{n}y_{i}}$$

(A14) $$NMSE=\frac{\sum_{i=1}^{n}\bar{{\left(y_{i}-x_{i}\right)}^{2}}}{\sum_{i=1}^{n}\bar{y_{i}x_{i}}}$$

(A15) $$NRMSE=\frac{\mathrm{RMSE}}{x_{\max}-x_{\min}}$$

(A16) $$PA=\sum_{i=1}^{n}\frac{\sum_{i=1}^{n}{\left(y_{i}-\bar{y}\right)}^{2}}{\left(n-1\right)\sigma \left(x_{i}\right)\sigma \left(y_{i}\right)}$$

(A17) $$R=\frac{\sum_{i=1}^{n}\left(x_{i}-\bar{x}\right)\left(y_{i}-\bar{y}\right)}{\sqrt{\sum_{i=1}^{n}{\left(x_{i}-\bar{x}\right)}^{2}}\cdot \sqrt{\sum_{i=1}^{n}{\left(y_{i}-\bar{y}\right)}^{2}}}$$

(A18) $$R^{2}=\frac{\sum_{i=1}^{n}{\left(y_{i}-\bar{y}\right)}^{2}}{\sum_{i=1}^{n}{\left(x_{i}-\bar{y}\right)}^{2}}$$

(A19) $$RE=\frac{\sum_{i=1}^{n}\left|x_{i}-y_{i}\right|}{\sum_{i=1}^{n}x_{i}}\cdot 100\%$$

(A20) $$RMS=\sqrt{\frac{1}{n}\sum_{i=1}^{n}x_{i}^{2}}$$

(A21) $$RMSE=\sqrt{\frac{1}{n}\sum_{i=1}^{n}{\left(x_{i}-y_{i}\right)}^{2}}$$

(A22) $$SEP=\frac{100}{\bar{x}}\mathrm{RMSE}$$

(A23) $$SMSE=\frac{1}{n}\frac{\sum_{i=1}^{n}{\left(x_{i}-y_{i}\right)}^{2}}{\sigma_{y}^{2}}$$

(A24) $$SOS=\sum_{i=1}^{n}{\left(y_{i}-x_{i}\right)}^{2}$$

(A25) $$SSE=\sum_{i=1}^{n}{\left(x_{i}-y_{i}\right)}^{2}$$
